# Hyperphosphorylation of BCL-2 family proteins underlies functional resistance to venetoclax in lymphoid malignancies

**DOI:** 10.1172/JCI170169

**Published:** 2023-11-15

**Authors:** Stephen Jun Fei Chong, Fen Zhu, Olga Dashevsky, Rin Mizuno, Jolin X.H. Lai, Liam Hackett, Christine E. Ryan, Mary C. Collins, J. Bryan Iorgulescu, Romain Guièze, Johany Penailillo, Ruben Carrasco, Yeonjoo C. Hwang, Denise P. Muñoz, Mehdi Bouhaddou, Yaw Chyn Lim, Catherine J. Wu, John N. Allan, Richard R. Furman, Boon Cher Goh, Shazib Pervaiz, Jean-Philippe Coppé, Constantine S. Mitsiades, Matthew S. Davids

**Affiliations:** 1 Department of Medical Oncology, Dana-Farber Cancer Institute, Harvard Medical School, Boston, Massachusetts, USA.; 2 Department of Physiology, Yong Loo Lin School of Medicine, National University of Singapore, Singapore.; 3 Department of Pathology, Brigham and Women’s Hospital, Boston, Massachusetts, USA.; 4 Helen Diller Family Comprehensive Cancer Center, UCSF, San Francisco, California, USA.; 5 Department of Microbiology, Immunology and Molecular Genetics, UCLA, Los Angeles, California, USA.; 6 Cancer Science Institute, National University of Singapore, Singapore.; 7 Division of Hematology and Medical Oncology, Weill Cornell Medicine, New York, New York, USA.

**Keywords:** Hematology, Oncology, Apoptosis survival pathways, Phosphoprotein phosphatases, Protein kinases

## Abstract

The B cell leukemia/lymphoma 2 (BCL-2) inhibitor venetoclax is effective in chronic lymphocytic leukemia (CLL); however, resistance may develop over time. Other lymphoid malignancies such as diffuse large B cell lymphoma (DLBCL) are frequently intrinsically resistant to venetoclax. Although genomic resistance mechanisms such as *BCL2* mutations have been described, this probably only explains a subset of resistant cases. Using 2 complementary functional precision medicine techniques — BH3 profiling and high-throughput kinase activity mapping — we found that hyperphosphorylation of BCL-2 family proteins, including antiapoptotic myeloid leukemia 1 (MCL-1) and BCL-2 and proapoptotic BCL-2 agonist of cell death (BAD) and BCL-2 associated X, apoptosis regulator (BAX), underlies functional mechanisms of both intrinsic and acquired resistance to venetoclax in CLL and DLBCL. Additionally, we provide evidence that antiapoptotic BCL-2 family protein phosphorylation altered the apoptotic protein interactome, thereby changing the profile of functional dependence on these prosurvival proteins. Targeting BCL-2 family protein phosphorylation with phosphatase-activating drugs rewired these dependencies, thus restoring sensitivity to venetoclax in a panel of venetoclax-resistant lymphoid cell lines, a resistant mouse model, and in paired patient samples before venetoclax treatment and at the time of progression.

## Graphical abstract

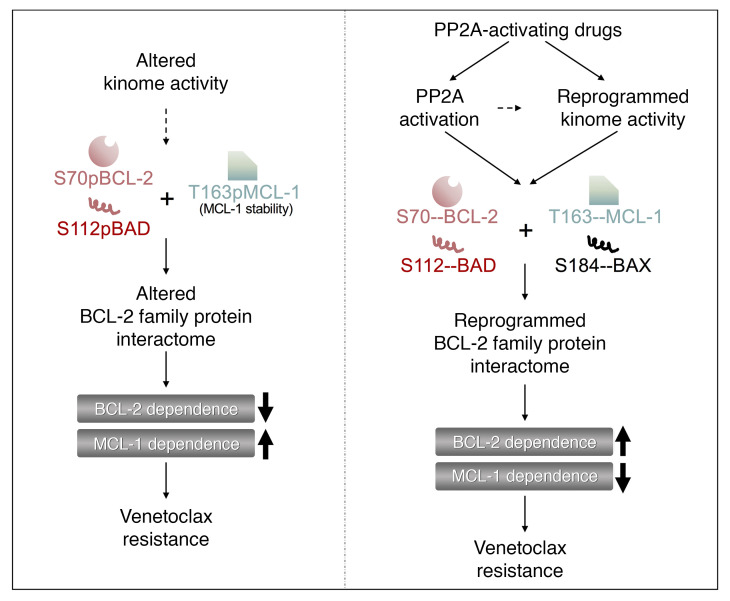

## Introduction

The incorporation of the B cell leukemia/lymphoma 2 (BCL-2) inhibitor venetoclax into the treatment paradigm for chronic lymphocytic leukemia (CLL) and acute myeloid leukemia (AML) has revolutionized the treatment of these diseases (1, 2). However, this therapy is not thought to be curative, and with longer-term follow-up, resistance has been increasingly recognized as a major challenge. Furthermore, common lymphoid malignancies such as diffuse large B cell lymphoma (DLBCL) are frequently intrinsically resistant to venetoclax (3). Thus, there is an urgent need to identify potential resistance mechanisms and alternative therapeutic approaches to overcome them. While acquired venetoclax resistance is explained in part by genetic mechanisms, such as the *BCL2* p.G101V that reduces binding of venetoclax to BCL-2, these mutations are only observed in about half of the patients with CLL on long-term venetoclax therapy, and when these mutations do occur, they are often found at a low variant allele frequency (VAF) (4–6). Thus, the venetoclax resistance mechanism(s) in 2 of the most common lymphoid malignancies (CLL and DLBCL) remain incompletely understood, particularly with regard to functional (i.e., nongenetic) resistance mechanisms.

Prior studies have suggested that posttranslational modification(s) of the BCL-2 family proteins could alter their functions in governing the intrinsic apoptotic pathway. In particular, phosphorylation of antiapoptotic BCL-2 at serine 70 (S70pBCL-2) has been shown to promote cancer cell survival in response to proapoptotic stimuli and to enhance its sequestration of the proapoptotic effector BAX (7–12). Phosphorylation of the antiapoptotic protein myeloid leukemia 1 (MCL-1) at threonine 163 (T163pMCL-1) has been shown to increase MCL-1 protein stability, thereby increasing the sequestration of proapoptotic proteins and inducing chemoresistance in lymphoid malignancies (13–16). In contrast, phosphorylation of BCL-2 agonist of cell death (BAD) at serine 112 (S112pBAD) or BCL-2 associated X, apoptosis regulator (BAX) at serine 184 (S184pBAX) inhibits its proapoptotic function (17–20). Given their relevance in promoting survival and chemoresistance, we hypothesized that increased S70pBCL-2, T163pMCL-1, S112pBAD, and/or S184pBAX may be present in resistant tumor cells from patients with lymphoid malignancies and, if so, may be an important factor in driving venetoclax resistance.

The protein phosphatase 2A (PP2A) is a serine/threonine phosphatase that dephosphorylates its target proteins. Numerous studies have demonstrated a tumor suppressor role of PP2A, and PP2A inactivation contributes to disease progression in several hematologic malignancies (12, 21–26). Recent work has linked PP2A inactivation to increased S70pBCL-2 and suggested a strong association between S70pBCL-2 and poor prognosis in certain lymphomas (12). Similarly, the stability and function of MCL-1, BAD, and BAX are governed in part by PP2A (18, 19, 27–30). Thus, we hypothesized that drugs that activate PP2A could reverse venetoclax resistance via dephosphorylation of BCL-2, MCL-1, BAD, and BAX, thereby resensitizing tumor cells to BCL-2 inhibition.

Here, we report data comparing venetoclax-sensitive with venetoclax-resistant malignant lymphoid cells, including a panel of cell lines, a lymphoid mouse model, and primary samples from patients with CLL before venetoclax treatment and at the time of progression while on venetoclax. We found that hyperphosphorylated BCL-2 family proteins were a crucial mechanism of intrinsic and acquired venetoclax resistance in lymphoid malignancies and we provide evidence supporting future clinical investigation of a therapeutic strategy utilizing PP2A-activating drugs (PADs) to resensitize resistant cells to venetoclax.

## Results

### BCL-2 family protein phosphorylation contributes to intrinsic and acquired venetoclax resistance in lymphoid malignancies.

**Figure 1:**
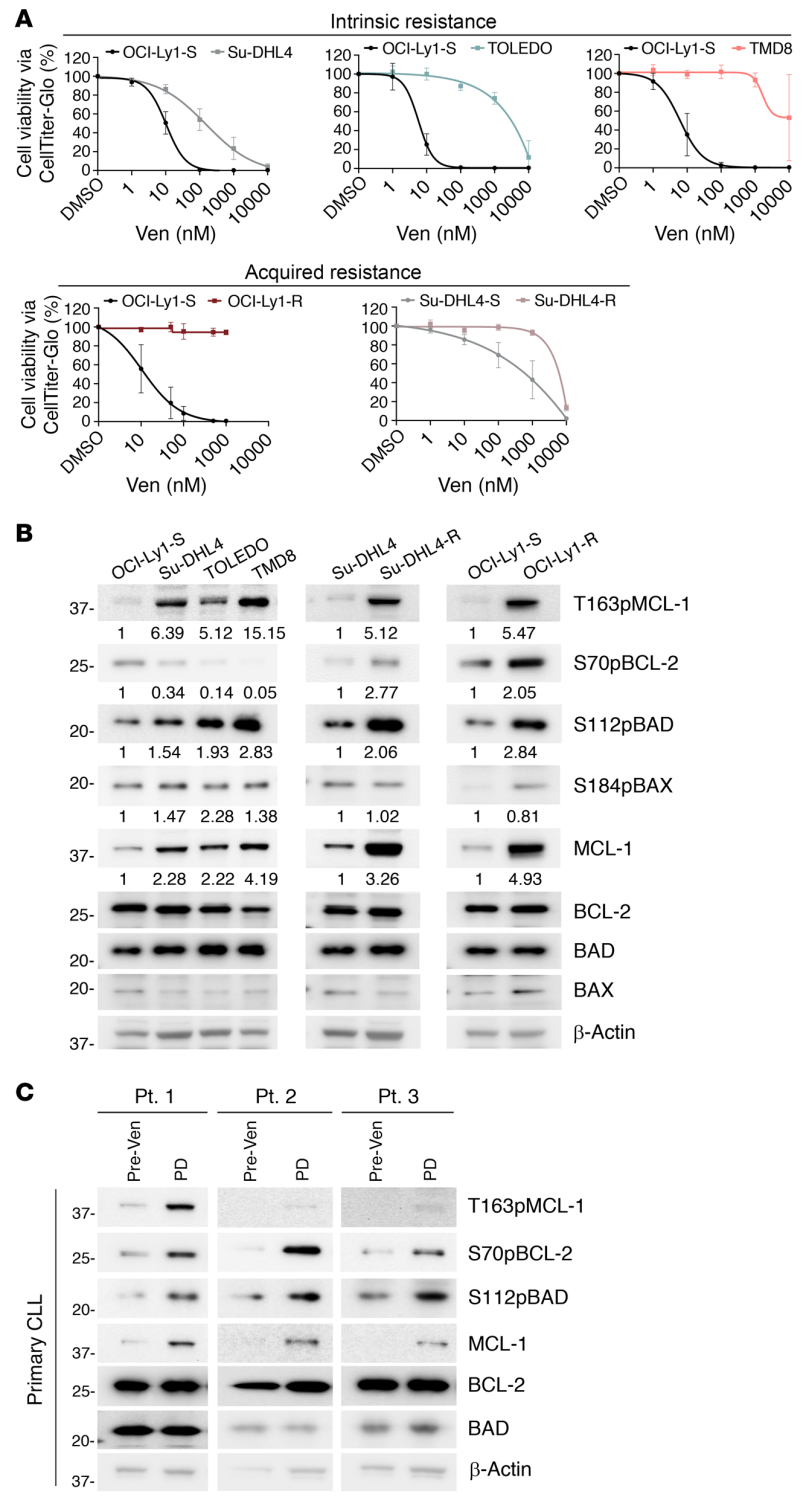
Venetoclax-resistant malignant lymphoid cells display hyperphosphorylated BCL-2 family proteins. (**A**) Cell viability of intrinsically resistant Su-DHL4 (*n* = 3), TOLEDO (*n* = 4), and TMD8 (*n* = 4) cells or acquired-resistance OCI-Ly1-R (*n* = 3) and Su-DHL4-R (*n* = 3) cells in comparison with OCI-Ly1-S or Su-DHL4 cells following treatment with increasing concentrations of ABT199/venetoclax (Ven) at 48 hours, measured by CTG assay. (**B**) Western blots showing increased T163pMCL-1, S70pBCL-2, S112pBAD, and MCL-1 levels in intrinsically resistant and acquired-resistance malignant lymphoid cells. Bands were quantified by ImageJ software (NIH). Normalized expression values derived from T163pMCL-1/β-actin, S70pBCL-2/BCL-2, S112pBAD/BAD, and MCL-1/β-actin are displayed below the targets in this and subsequent specific figures. (**C**) Western blots showing increased T163pMCL-1 (*n* = 8), S70pBCL-2 (*n* = 9), S112pBAD (*n* = 6), and MCL-1 (*n* = 9) levels in primary CLL samples from patients on venetoclax with progressive disease (PD) compared with paired pre-venetoclax primary CLL patient samples (human, in vivo). Quantification is displayed in Supplemental Figure 1C. The sample number is different due to sample availability. PT, patient.

To evaluate the potential contribution of BCL-2 family protein phosphorylation to venetoclax resistance, we examined lymphoid malignant cell lines that were intrinsically sensitive and resistant to venetoclax. We first identified 3 DLBCL cell lines — double-hit Su-DHL4, germinal center B cell TOLEDO, and activated B cell TMD8 — that were intrinsically resistant to venetoclax in relation to the intrinsically sensitive DLBCL cell line OCI-Ly1-S (Figure 1A). We also used 2 cell lines with acquired venetoclax resistance, generated from their parental OCI-Ly1-S and Su-DHL4 (OCI-Ly1-R, Su-DHL4-R) lines (Figure 1A).

Next, we measured the phosphorylation status of the BCL-2 family members to evaluate for an association between their phosphorylation levels and resistance to venetoclax. We observed a selective increase in T163pMCL-1 (antiapoptotic activation) and S112pBAD (proapoptotic inhibition) in intrinsically resistant Su-DHL4, TOLEDO, and TMD8 cells, and a further increase in T163pMCL-1, S70pBCL-2 (antiapoptotic activation), and S112pBAD in the acquired-resistance Su-DHL4-R and OCI-Ly1-R lines. There was, however, no consistent increase in S184pBAX (proapoptotic inhibition) (Figure 1B). These findings suggest that the intrinsically higher T163pMCL-1 and S112pBAD levels in Su-DHL4, TOLEDO, and TMD8 cells may have contributed to intrinsic venetoclax resistance, whereas a further increase in T163pMCL-1, S70pBCL-2, and S112pBAD levels was required for acquired venetoclax resistance following continuous exposure to venetoclax. We also observed an increase in MCL-1 protein levels across all intrinsic and acquired-resistance lines (Figure 1B), supporting previous observations of MCL-1 stabilization by T163pMCL-1 (13–15). No obvious changes in protein expression of PP2A subunits (B56/PP2A catalytic subunits) were observed in these cell lines (Supplemental Figure 1A; supplemental material available online with this article; https://doi.org/10.1172/JCI170169DS1).

We next utilized high-throughput kinase activity mapping (HT-KAM), a functional assay that detects the phosphorylation of 11 mer peptides corresponding to their protein phosphorylation sites by kinases from cell lysates and confirmed the increase in MCL-1, BCL-2, and BAD phosphorylation in both the intrinsically and acquired-resistance Su-DHL4 and OCI-Ly1-R, in comparison with OCI-Ly1-S (Supplemental Figure 1B). Importantly, we also observed significant increases in S70pBCL-2, T163pMCL-1, MCL-1, and S112pBAD levels, but not S184pBAX levels, in primary CLL patient samples at the time of progression while on venetoclax compared with their pre-venetoclax samples (Figure 1C and Supplemental Figure 1, C and D). These findings from primary CLL samples from patients treated with venetoclax support our hypothesis that BCL-2 family phosphorylation contributes to venetoclax resistance in lymphoid malignancies.

**Figure 2:**
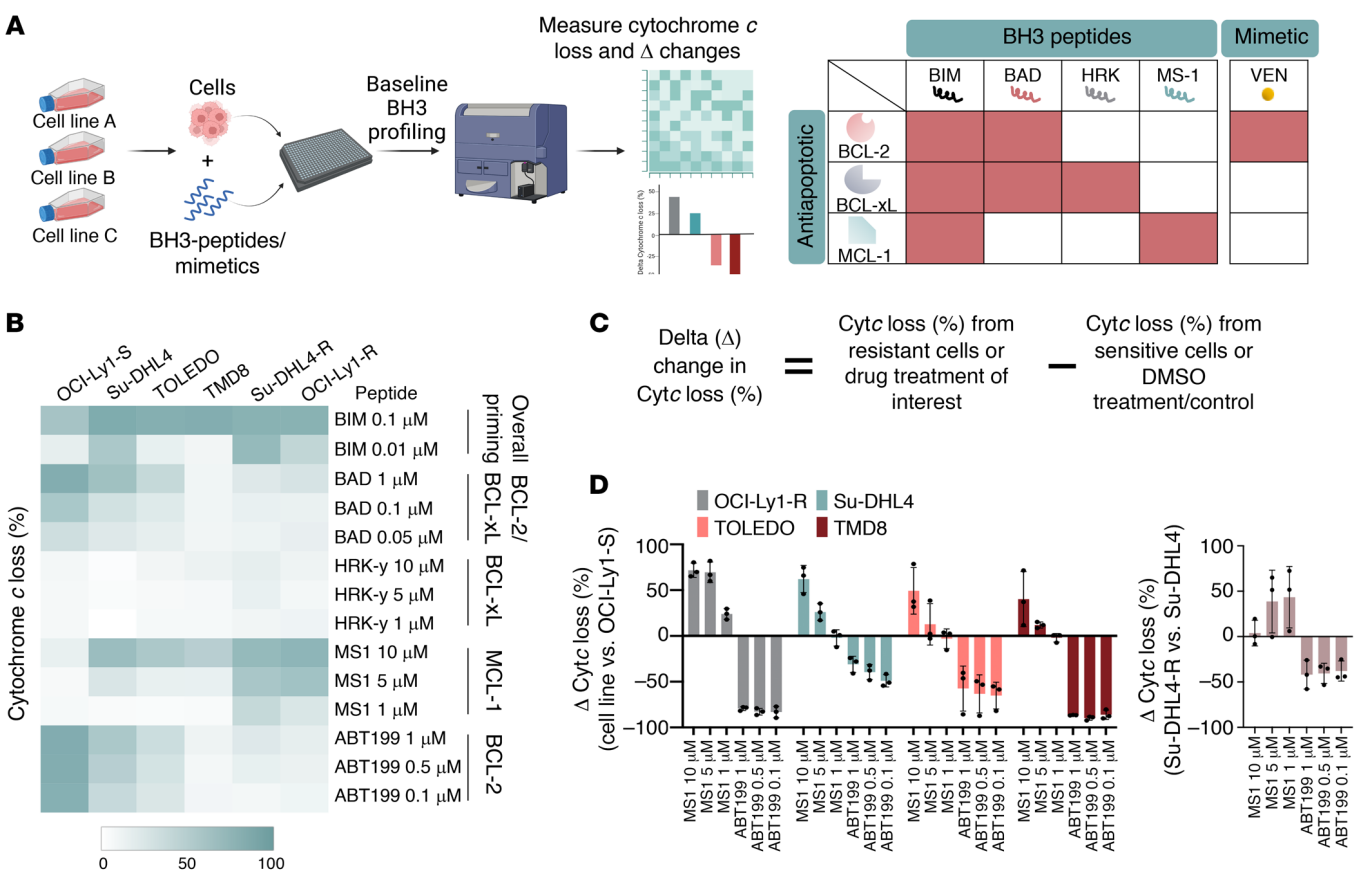
Venetoclax-resistant malignant lymphoid cells display increased MCL-1 dependence and decreased BCL-2 dependence. (**A**) Diagram showing the baseline BH3-profiling technique, in which cells are required to be incubated with BH3 peptides/mimetic to induce Cyt*c* release. Heatmap demonstrates the sensitivity/dependence of antiapoptotic BCL-2 family members toward their specific BH3 peptides/mimetic, as represented by the intensity of Cyt*c* release in red. Higher Cyt*c* release indicates a greater dependence on an antiapoptotic protein(s). The diagram was generated using BioRender software. (**B**) Baseline BH3 profiling of malignant lymphoid cell lines, depicted by a heatmap of Cyt*c* loss intensity following individual BH3 peptides or ABT199/venetoclax incubation (*n* = 3). Higher Cyt*c* release by MS1 indicates MCL-1 dependence, and lower Cyt*c* release by BAD or venetoclax/ABT199 indicates lower BCL-2 dependence. (**C**) Delta (Δ) changes in the percentage of Cyt*c* loss were calculated by deducting the percentage of Cyt*c* loss of sensitive cell lines from that of resistant cell lines or the percentage of Cyt*c* loss of vehicle-treated cells from that of drug-treated cells. (**D**) Δ Changes in the percentage of Cyt*c* loss indicate an increase in MCL-1 dependency and a drop in BCL-2 dependency. A positive Δ change in the percentage indicates increased net Cyt*c* loss, and a negative Δ change in the percentage indicates decreased net Cyt*c* loss.

Sensitivity to BH3 mimetics, such as venetoclax, is directly related to the specific survival dependence of cancer cells on antiapoptotic BCL-2 family proteins. To evaluate antiapoptotic dependencies, we used the functional BH3 profiling technique, which relies on the selective binding of BH3-only peptides or BH3 mimetics to antiapoptotic proteins to induce cytochrome *c* (Cyt*c*) release, thereby identifying the antiapoptotic protein(s) the cell functionally depends on for survival (Figure 2A) (31). Indeed, we observed higher MCL-1 dependence (MS-1 peptides) and lower BCL-2 dependence (BAD peptides and ABT199) across the intrinsically resistant lymphoid lines in comparison with OCI-Ly1-S (Figure 2B). In particular, we detected a clear switch of dependence from BCL-2 to MCL-1 in OCI-Ly1-R and Su-DHL4-R cells compared with their parental lines (Figure 2B and Supplemental Figure 1E). The delta (Δ) changes in Cyt*c* loss between these resistant and sensitive cell lines further indicated a substantial increase in MCL-1 dependence and a concomitant decrease in BCL-2 dependence (Figure 2, C and D). These findings suggest that the cells were intrinsically resistant to venetoclax at least in part because of dependence on antiapoptotic protein(s) other than BCL-2, or may have acquired venetoclax resistance by switching dependence from BCL-2 to a different antiapoptotic protein.

**Figure 3:**
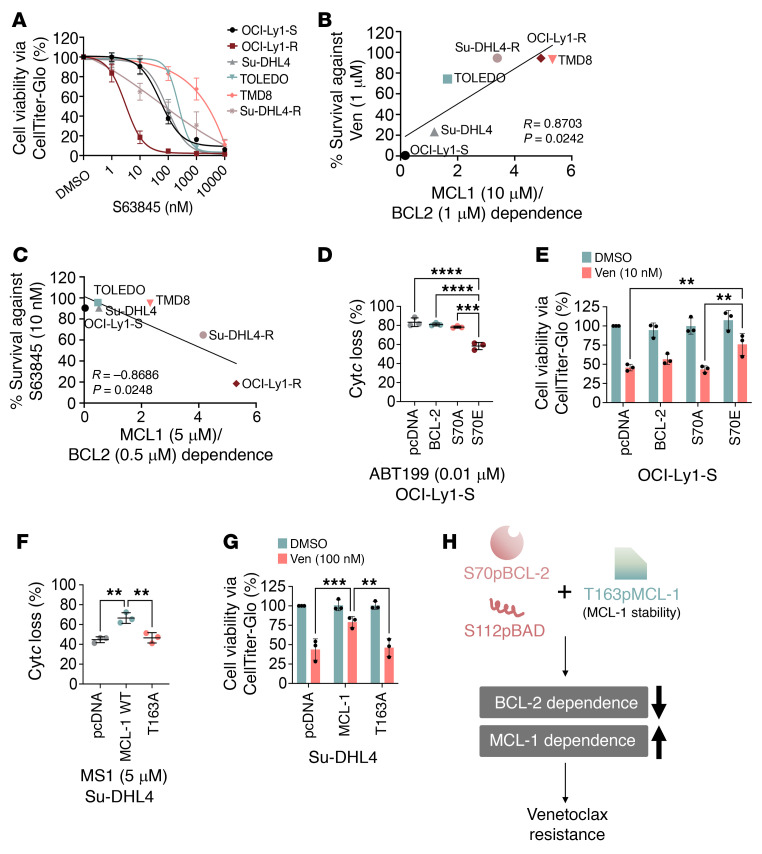
Changes in antiapoptotic protein dependencies are required for venetoclax resistance. (**A**) Viability of OCI-Ly1-R, Su-DHL4-R, Su-DHL4, TOLEDO, TMD8, and OCI-Ly1-S cells following treatment with increasing concentrations of S63845 (MCL-1 inhibitor), measured by CTG assay. Tukey’s multiple-comparison test was used. (**B**) Pearson’s correlation between survival percentage against ABT199/venetoclax treatment and MCL-1/BCL-2 dependence ratio. Cells with high MCL-1 but low BCL-2 dependence survived better against venetoclax. (**C**) Pearson’s correlation between percentage survival against S63845 treatment and MCL-1/BCL-2 dependence ratio. Cells with low MCL-1 but high BCL-2 dependence survived better against S63845. (**D**) ABT199/venetoclax-induced Cyt*c* percentage loss following transient transfection with empty vector (pcDNA3.1), WT BCL-2, p.S70A, or p.S70E mutants in OCI-Ly1-S cells (*n* = 3). Tukey’s multiple-comparison test was used. (**E**) Cell viability of pcDNA3.1 (pcDNA), WT BCL-2, p.S70A (S70A), or p.S70E (S70E) transiently transfected OCI-Ly1-S cells following treatment with 10 nM ABT199/venetoclax for 48 hours (*n* = 3). Šidák’s multiple-comparison test was used. (**F**) MS1-induced Cyt*c* percentage loss following transient transfection with pcDNA3.1, WT MCL-1, or the p.T163 mutant in Su-DHL4 cells (*n* = 3). Dunnett’s multiple-comparison test was used. (**G**) Cell viability of pcDNA3.1, WT MCL-1 or p.T163A transient transfected Su-DHL4 cells following treatment with 100 nM ABT199/venetoclax for 48 hours (*n* = 3). Šidák’s multiple-comparison test was used. (**H**) Diagram showing that increased S70pBCL-2, T163pMCL-1, and S112pBAD induced a dependence switch from BCL-2 to MCL-1 for venetoclax resistance. The diagram was generated using Microsoft Powerpoint software. **P < 0.01, ***P < 0.001, and ****P < 0.0001.

We also observed that venetoclax-resistant cell lines such as OCI-Ly1-R and Su-DHL4-R became more sensitive to the selective MCL-1 inhibitor S63845, consistent with their greater functional dependence on MCL-1 (Figure 3A). This provides a proof of concept that a change in antiapoptotic dependence may lead to a change in sensitivity to the various BH3 mimetics. Moreover, we found that cell lines that possessed a high MCL-1 to BCL-2 dependence ratio were likely to be less sensitive to BCL-2 inhibition with venetoclax (Figure 3B), and more sensitive to MCL-1 inhibition with S63845 (Figure 3C). Our findings therefore suggest that sensitivity to venetoclax or S63845 in lymphoid malignancies relied critically on the interplay between BCL-2 and MCL-1 dependence.

To further evaluate whether these changes in BCL-2 and MCL-1 dependencies were mechanistically related to their protein phosphorylation, such as S70pBCL-2 and T163pMCL-1, we induced transient overexpression of the phosphomimetic (p.S70E) or nonphosphorylatable (p.S70A) mutants of S70pBCL-2 in OCI-Ly1-S cells (Supplemental Figure 1F). We observed a drop in BCL-2 dependence in cells with the p.S70E mutation but not in those with the p.S70A mutation (Figure 3D). These observations were consistent with the enhanced resistance to venetoclax observed in OCI-Ly1-S cells transfected with p.S70E (Figure 3E). In a reciprocal manner, overexpression of WT MCL-1 further increased MCL-1 dependence in Su-DHL4 cells but not in the nonphosphorylatable mutant of T163pMCL-1 (p.T163A), which destabilized the protein expression of MCL-1 (Figure 3F and Supplemental Figure 1G). These observations were again associated with increased venetoclax resistance for WT MCL-1 but not p.T163A (Figure 3G). Collectively, these findings demonstrate that enforced phosphorylation of BCL-2 family proteins triggered cells to mimic the resistant phenotypes of OCI-Ly1-R or Su-DHL4-R cells by switching their functional dependence from BCL-2 to MCL-1 (Figure 3H).

### Targeting BCL-2 family protein phosphorylation restores BCL-2 dependence.

Although MCL-1 inhibition effectively kills venetoclax-resistant cell lines that are functionally dependent on MCL-1, direct targeting of MCL-1 in patients has to date proven to be challenging because of cardiotoxicity (32, 33). Therefore, alternative approaches are needed to safely and effectively treat lymphoid malignancies that are resistant to venetoclax. On the basis of our initial experiments, we hypothesized that pharmacologically decreasing BCL-2 family protein phosphorylation would resensitize resistant cells to venetoclax. We previously demonstrated that a PAD known as FTY720 (fingolimod), an FDA-approved drug for multiple sclerosis (34), reduces S70pBCL-2 (11). Therefore, we next evaluated whether FTY720 could simultaneously reduce S70pBCL-2, T163pMCL-1, S112pBAD, and S184pBAX in venetoclax-resistant cells and thereby rewire their dependence on BCL-2.

**Figure 4:**
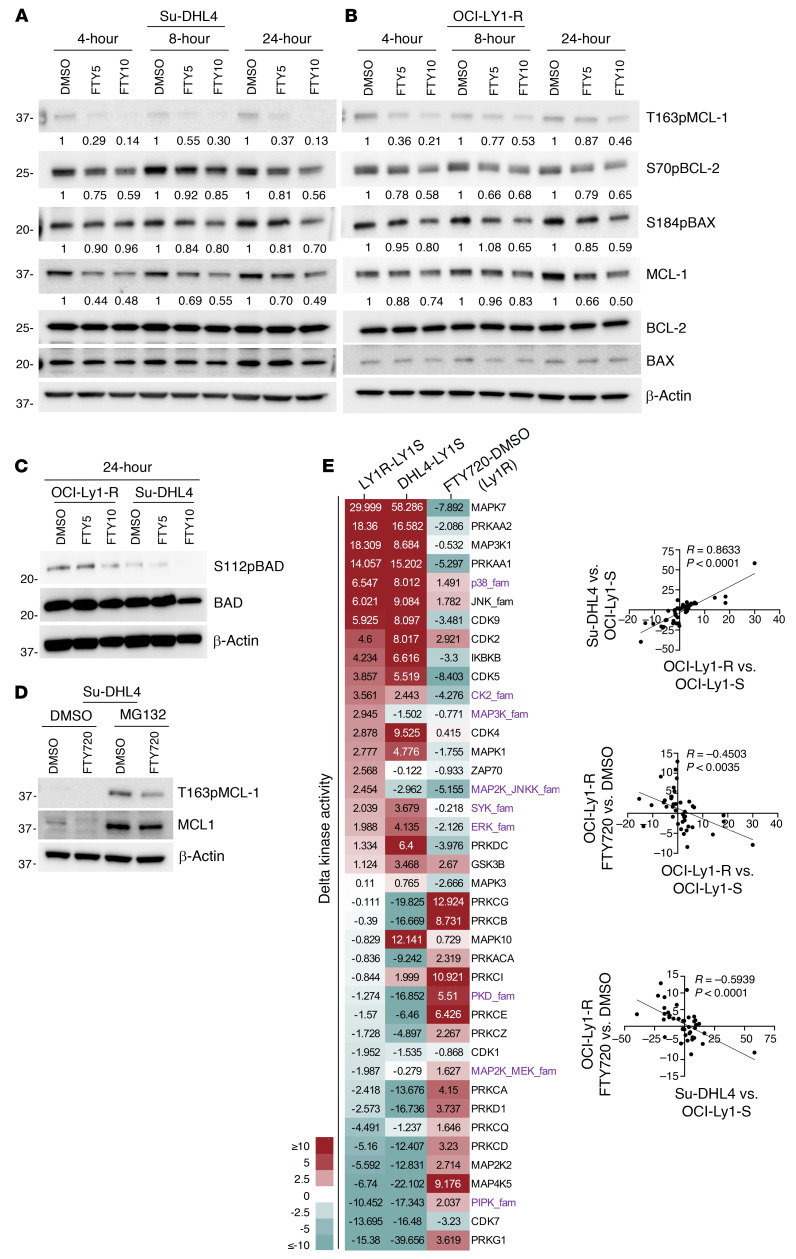
The phosphatase activator FTY720 reduces BCL-2 family protein phosphorylation. (**A** and **B**) Western blots showing the reduction of T163pMCL-1, S70pBCL-2, S184pBAX, and MCL-1 in Su-DHL4 and OCI-Ly1-R cells following treatment with FTY720 (5–10 μM) for 4, 8, and 24 hours. (**C**) Western blots showing the reduction of S112pBAD in Su-DHL4 and OCI-Ly1-R cells following treatment with 5–10 μM FTY720 (FTY5 and FTY10) for 24 hours. (**D**) Western blots showing the reduction of T163pMCL-1 but not MCL-1 in Su-DHL4 cells following pretreatment with the proteasomal inhibitor MG132 (5 μM) for 4 hours and treatment with FTY720 (10 μM) for 4 hours. (**E**) Δ Kinase enzymatic activities of OCI-Ly1-R minus OCI-Ly1-S, Su-DHL4 minus OCI-Ly1-S, and OCI-Ly1-R treated with FTY720 minus DMSO, measured by HT-KAM assay. Pearson’s correlation coefficient *R* and *P* values were measured between the different Δ kinase enzymatic activity columns.

Increasing doses of FTY720 at various time points showed a clear reduction of S70pBCL-2, T163pMCL-1, total MCL-1, and S184pBAX in all resistant cell lines (Figure 4, A and B, and Supplemental Figure 2, A and B). Similarly, S112pBAD was decreased in these resistant cell lines (Figure 4C). To rule out the possibility that the reduction in T163pMCL-1 was due to a reduction in total MCL-1 protein levels, we prevented MCL-1 degradation by inhibiting the proteasome with MG132 and observed that FTY720 still reduced T163pMCL-1 (Figure 4D). FTY720 thus likely reduced T163pMCL-1 prior to destabilizing MCL-1.

Since several kinases are known to phosphorylate BCL-2 family proteins (7), we used the HT-KAM assay to evaluate whether changes in the BCL-2 family protein phosphorylation seen with acquired venetoclax resistance are further coordinated by changes in the kinome enzymatic activity landscape. We specifically chose to assess the activity of kinases and kinase families (MAPKs, PKA/C/D, CDKs, and AMPK) known to include at least 1 documented member that could affect the phosphorylation and stability of the BCL-2 family (7, 13–15, 35–40). We observed a significant change in the kinome enzymatic activity landscape following acquisition of venetoclax resistance in OCI-Ly1-R cells compared with their parental OCI-Ly1-S cells (Figure 4E). This altered kinome enzymatic activity landscape in the OCI-Ly1-R cells mimicked that of the intrinsically venetoclax-resistant Su-DHL4 cells (Figure 4E, *R* = 0.8633, *P* < 0.0001), thus suggesting that venetoclax resistance is associated with a distinct pattern of altered kinome activity. Strikingly, we observed a clear reprogramming of the kinome enzymatic activity following FTY720 treatment, reverting the resistant kinome back to a sensitive state (Figure 4E, *R* = –0.4503, *P* < 0.0035 and *R* = –0.5939, *P* < 0.0001). We also independently verified the activity of some kinases by confirming the inactivation of AKT (reduced Ser473 phosphorylation), downstream activation of GSK3β (reduced Ser9 phosphorylation) and inactivation of ERK (reduced Thr202/Tyr204 phosphorylation) in both the acquired and intrinsic venetoclax-resistant cell lines (Supplemental Figure 2C).

**Figure 5:**
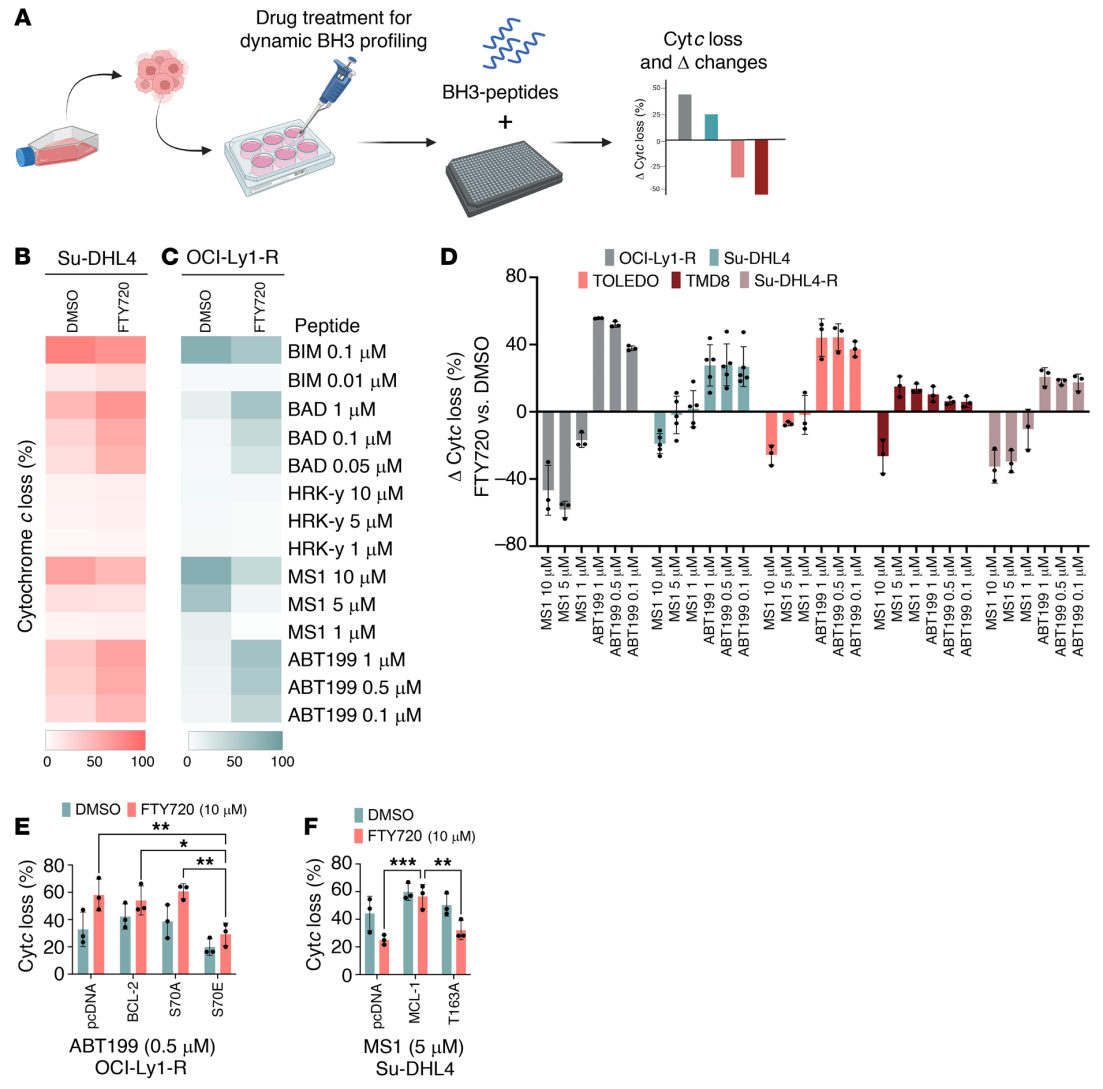
Reduction in BCL-2 family protein phosphorylation rewires resistant cells to BCL-2 dependence. (**A**) Diagram demonstrating the DBP technique. Cells were treated with the drug of interest prior to BH3 profiling. DBP identifies whether a drug of interest changes the antiapoptotic dependence(ies) of cells. The diagram was generated using BioRender software. (**B** and **C**) DBP of Su-DHL4 (*n* = 5) and OCI-Ly1-R (*n* = 3) cells following treatment with FTY720 (10 μM) for 4 hours. (**D**) Δ Changes in the percentage of Cyt*c* loss between FTY720 and DMSO treatments in the acquired-resistance and intrinsically resistant cell lines (*n* = 5 for Su-DHL4, *n* = 3 for all other cell lines). (**E**) Percentage of Cyt*c* loss with ABT199/venetoclax (0.5 μM) following a 4-hour treatment with FTY720 in pcDNA3.1-, WT BCL-2–, p.S70A-, or p.S70E-transfected OCI-Ly1-R cells (*n* = 3). Šidák’s multiple-comparison test was used. (**F**) Percentage of Cyt*c* loss with MS1 peptide (5 μM) following a 4-hour FTY720 treatment in pcDNA3.1-, WT MCL-1–, or p.T163A-transfected Su-DHL4 cells (*n* = 3). Dunnett’s multiple-comparison test was used. *P < 0.05, **P < 0.01, and ***P < 0.001.

To measure the antiapoptotic dependence of the venetoclax-resistant cells following treatment with FTY720, we used dynamic BH3 profiling (DBP), a variation of BH3 profiling that measures the net effect of ex vivo drug treatments on the antiapoptotic dependence of cells (Figure 5A). Treatment with FTY720 was capable of rewiring dependence from MCL-1 to BCL-2 in all resistant lymphoid cell lines (Figure 5, B and C, and Supplemental Figure 3, A–C), consistently showing a net decrease in MCL-1 dependence and a reciprocal increase in BCL-2 dependence (Figure 5D).

To confirm that these changes in dependencies were governed by the reduction of BCL-2 family protein phosphorylation, we induced transient overexpression of the mutants of S70pBCL-2 and T163pMCL-1 in OCI-Ly1-R and Su-DHL4 cells, respectively. Indeed, the p.S70E, but not p.S70A, mutant prevented the increase in BCL-2 dependence following FTY720 treatment, while WT MCL-1, but not the p.T163A mutant, prevented the decrease in MCL-1 dependence following FTY720 treatment (Figure 5, E and F, and Supplemental Figure 3D). These observations were corroborated by the diminished effect of FTY720 in reducing phosphorylation of S70pBCL-2 and T163pMCL-1 in p.S70E-mutant and WT MCL-1–transfected cells, respectively (Supplemental Figure 3, E and F). These findings thus suggest that the rewiring of dependence from MCL-1 to BCL-2 following treatment with FTY720 was directly related to reducing phosphorylation of BCL-2 family proteins. We also noted that FTY720 could still destabilize MCL-1 protein in p.T163A-mutant–transfected cells, even when p.T163A did not have a phosphate group (Supplemental Figure 3F). This was likely due to the inactivation of AKT, which led to the downstream activation of GSK3β (Supplemental Figure 2C) and subsequent phosphorylation of S159pMCL-1 (Supplemental Figure 3G), which tags MCL-1 for proteasomal degradation (Supplemental Figure 3H) (38).

### Changes in survival dependence are governed by an altered apoptotic protein interactome.

**Figure 6:**
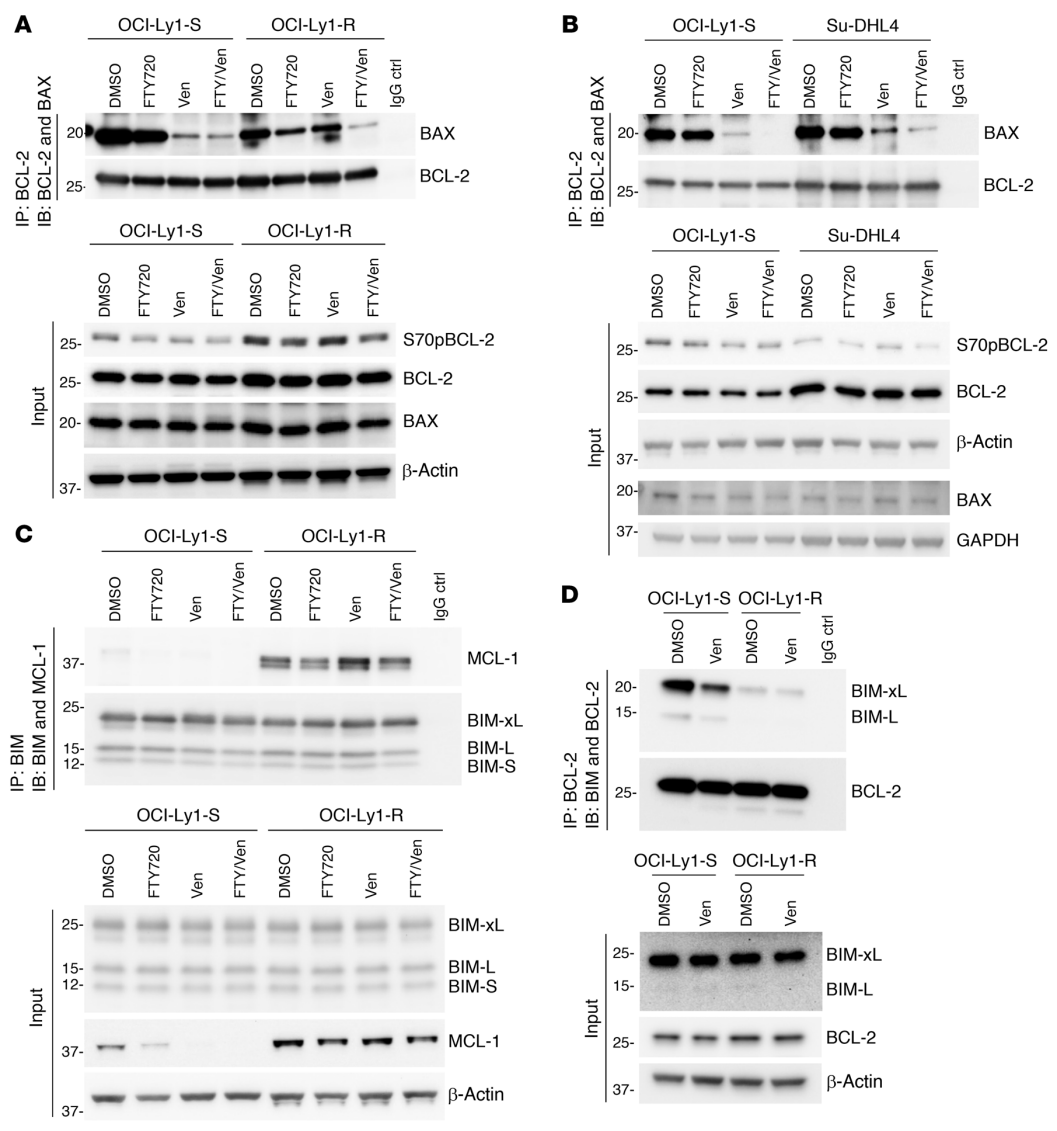
Changes in antiapoptotic dependencies involve the disruption of pro- and antiapoptotic protein interactions. (**A** and **B**) Co-IP of BCL-2 and immunoblotting (IB) of BAX and BCL-2 in OCI-Ly1-R, Su-DHL4, and OCI-Ly1-S cells following a 4-hour pretreatment with FTY720 (10 μM) and a subsequent 4-hour cotreatment with ABT199/venetoclax (100 nM). The venetoclax concentration was based on the lowest effective dose used for BH3 profiling. Input shows S70pBCL-2, BCL-2, BAX, β-actin, and/or GAPDH. The same sample for input in Figure 6B was run in 2 separate gels. (**C**) Co-IP of BIM isoforms and immunoblots of MCL-1 and BIM isoforms in OCI-Ly1-R and OCI-Ly1-S cells following a 4-hour pretreatment with FTY720 (10 μM) and a subsequent 4-hour cotreatment with ABT199/venetoclax (100 nM). Input shows BIM isoforms, MCL-1, and β-actin. (**D**) Co-IP of BCL-2 and immunoblots of BIM isoforms and BCL-2 in OCI-Ly1-R and OCI-Ly1-S cells following a 4-hour treatment with ABT199/venetoclax (100 nM). Input shows BIM isoforms, BCL-2, and β-actin. Ctrl, control.

It was previously shown that S70pBCL-2 enhances the interaction between BCL-2 and BAX, while T163pMCL-1 stabilizes MCL-1, which in turn enhances the sequestration of BIM (9, 10, 13, 16, 41). Given that the mechanism of action of venetoclax is to bind to BCL-2 and dissociate BAX, thereby leading to mitochondrial pore formation, Cyt*c* release, and apoptosis, we next examined whether a mechanism underlying functional venetoclax resistance involves an altered interactome of apoptotic proteins. Indeed, we observed less effective dissociation of BAX from BCL-2 in OCI-Ly1-R or Su-DHL4 cells compared with OCI-Ly1-S when these cells were treated with venetoclax (Figure 6, A and B). The stronger interaction between BCL-2 and BAX also corresponded to higher S70pBCL-2 in the acquired-resistance OCI-Ly1-R cells (Figure 6A, input). As MCL-1 protein is more stabilized in OCI-Ly1-R cells, we observed a greater interaction between MCL-1 and all BIM isoforms (Figure 6C). Reciprocally, baseline interaction between BIM and BCL-2 was markedly reduced in OCI-Ly1-R cells as compared with OCI-Ly1-S cells (Figure 6D), therefore corroborating the heightened sequestration of BIM by increased MCL-1 expression (Figure 6C). Additionally, venetoclax could substantially reduce BIM and BCL-2 interaction in OCI-Ly1-S cells but not in OCI-Ly1-R cells, albeit the latter had lower interaction at baseline (DMSO) with potentially negligible functional consequences (Figure 6D). These observations suggest that hyperphosphorylated antiapoptotic proteins in resistant cells possess a higher capability of sequestering and thereby inhibiting proapoptotic proteins. These findings corroborate our baseline BH3-profiling results (Figure 2, B–D), as venetoclax-resistant cells were more sensitive to MS1 peptide and S63845 but not to BAD peptide and venetoclax because of their greater dependence on MCL-1.

**Figure 7:**
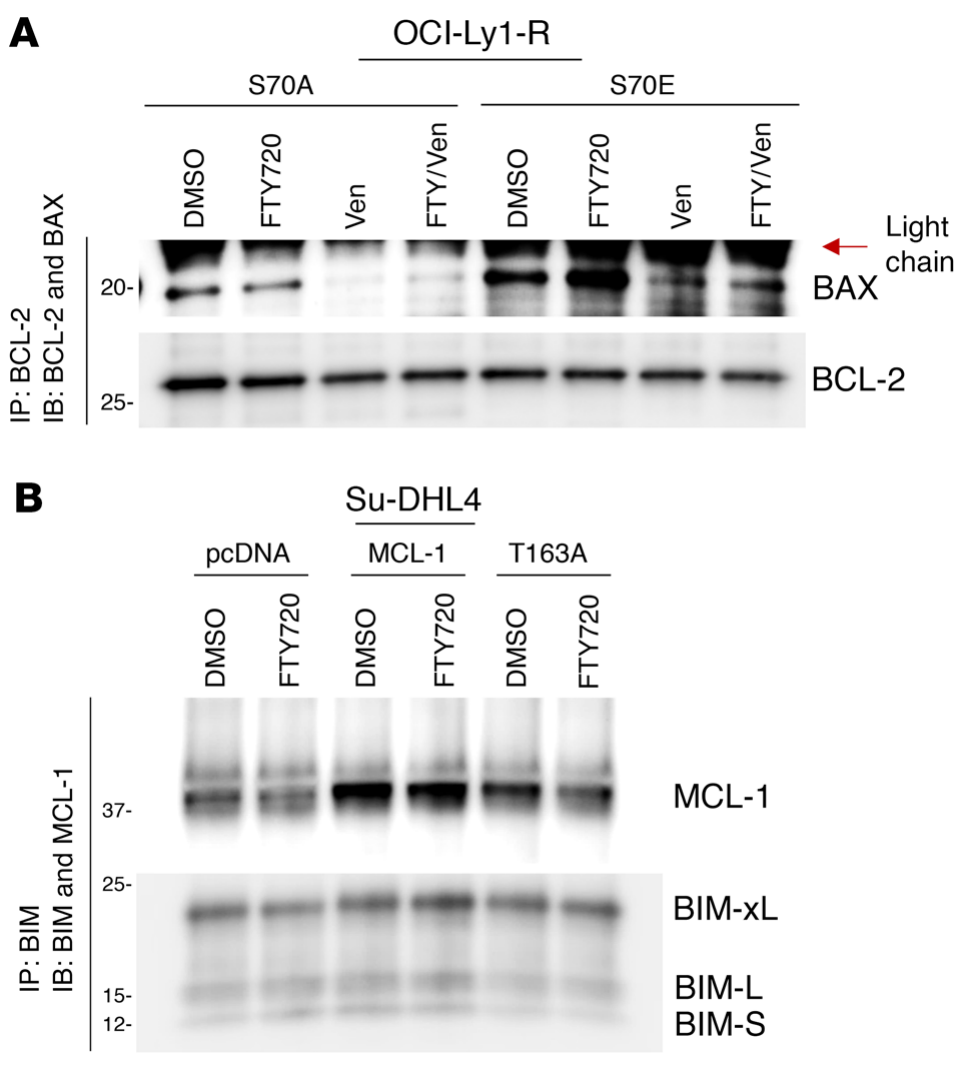
Changes in pro- and antiapoptotic protein interactions are governed by BCL-2 family protein phosphorylation. (**A**) Co-IP of BCL-2 and immunoblots of BAX and BCL-2 in p.S70A- or p.S70E-transfected OCI-Ly1-R cells following a 4-hour pretreatment with FTY720 (10 μM) and a subsequent 4-hour cotreatment with ABT199/venetoclax (100 nM). (**B**) Co-IP of BIM isoforms and immunoblots of MCL-1 and BIM isoforms in pcDNA3.1-, WT MCL-1–, or p.T163A-transfected Su-DHL4 cells following a 4-hour treatment with FTY720 (10 μM).

Importantly, FTY720 was able to modulate this resistant apoptotic interactome by reducing the interaction between BCL-2 and BAX as well as between MCL-1 and BIM, in conjunction with reduced S70pBCL-2, T163pMCL-1, and MCL-1 (Figure 6, A–C). Again, transient overexpression of p.S70E could resist the effects of FTY720 in dissociating BAX from BCL-2 (Figure 7A). Similarly, WT MCL-1 overexpression, which could partially prevent MCL-1 reduction by FTY720, allowed MCL-1 to still sequester BIM (Figure 7B). These findings improve our understanding of the mechanisms underlying the ability of FTY720 to restore the apoptotic interactome through a reduction of BCL-2 family protein phosphorylation, thereby rewiring venetoclax-resistant cells from MCL-1 dependence to BCL-2 dependence.

### Restoring BCL-2 dependence resensitizes resistant cells to venetoclax.

**Figure 8:**
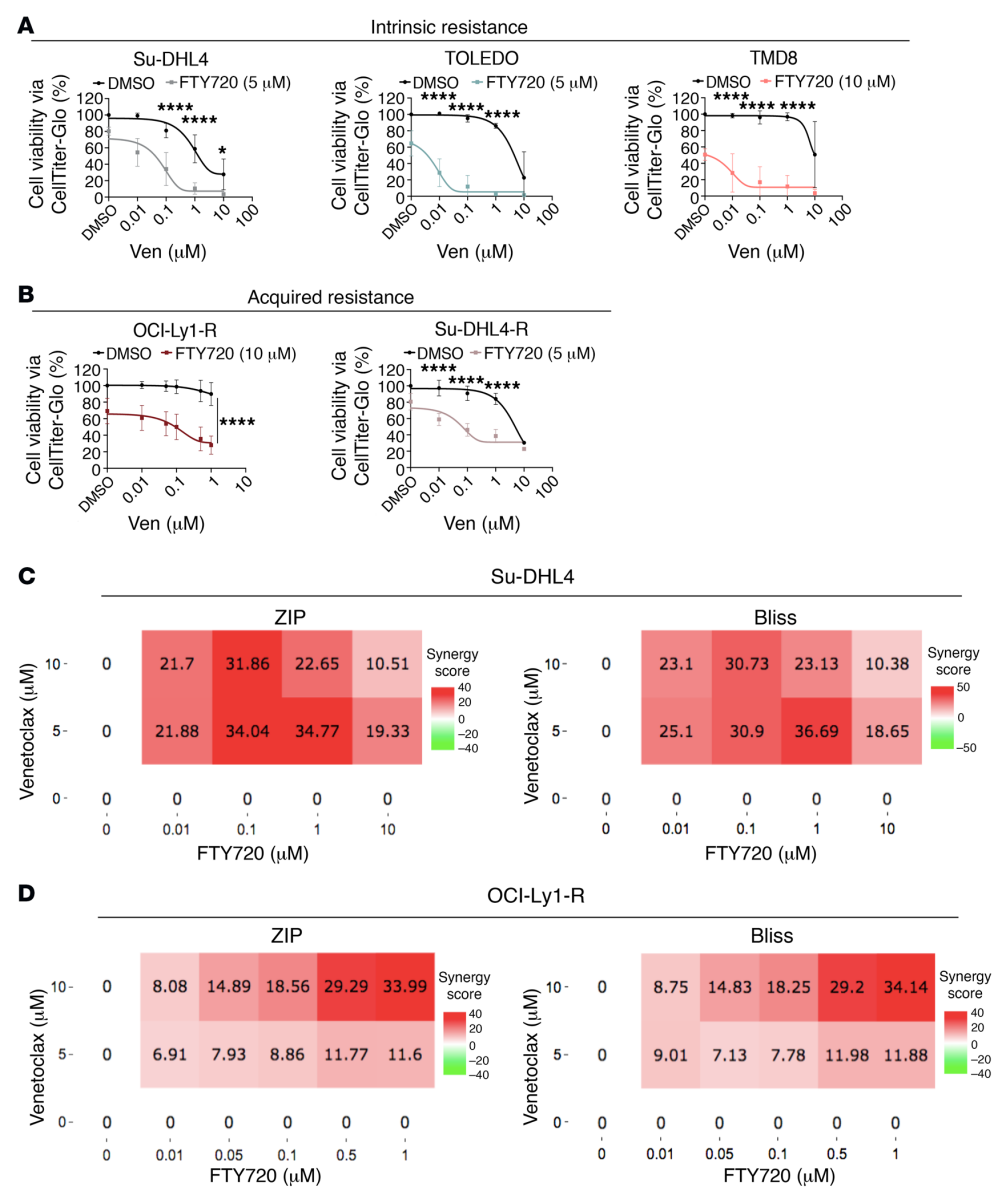
Treatment with FTY720 resensitizes resistant cells to venetoclax. (**A** and **B**) Viability of Su-DHL4 (*n* = 6), TOLEDO (*n* = 3), and TMD8 (*n* = 4) cells or OCI-Ly1-R (*n* = 6) cells following a 4-hour pretreatment with FTY720 and subsequent 48 hours of increasing concentrations of cotreatment with ABT199/venetoclax or Su-DHL4-R (*n* = 3) following a 4-hour pretreatment with FTY720 and 24 hours of increasing concentrations of cotreatment with ABT199/venetoclax, measured by CTG assay. Tukey’s multiple-comparison tests were used. *P < 0.05 and ****P < 0.0001. (**C** and **D**) Synergy scores for Su-DHL4 and OCI-Ly1-R cells were computed by SynergyFinder based on ZIP and Bliss Synergy models. Red indicates synergism and green indicates antagonism.

**Figure 9:**
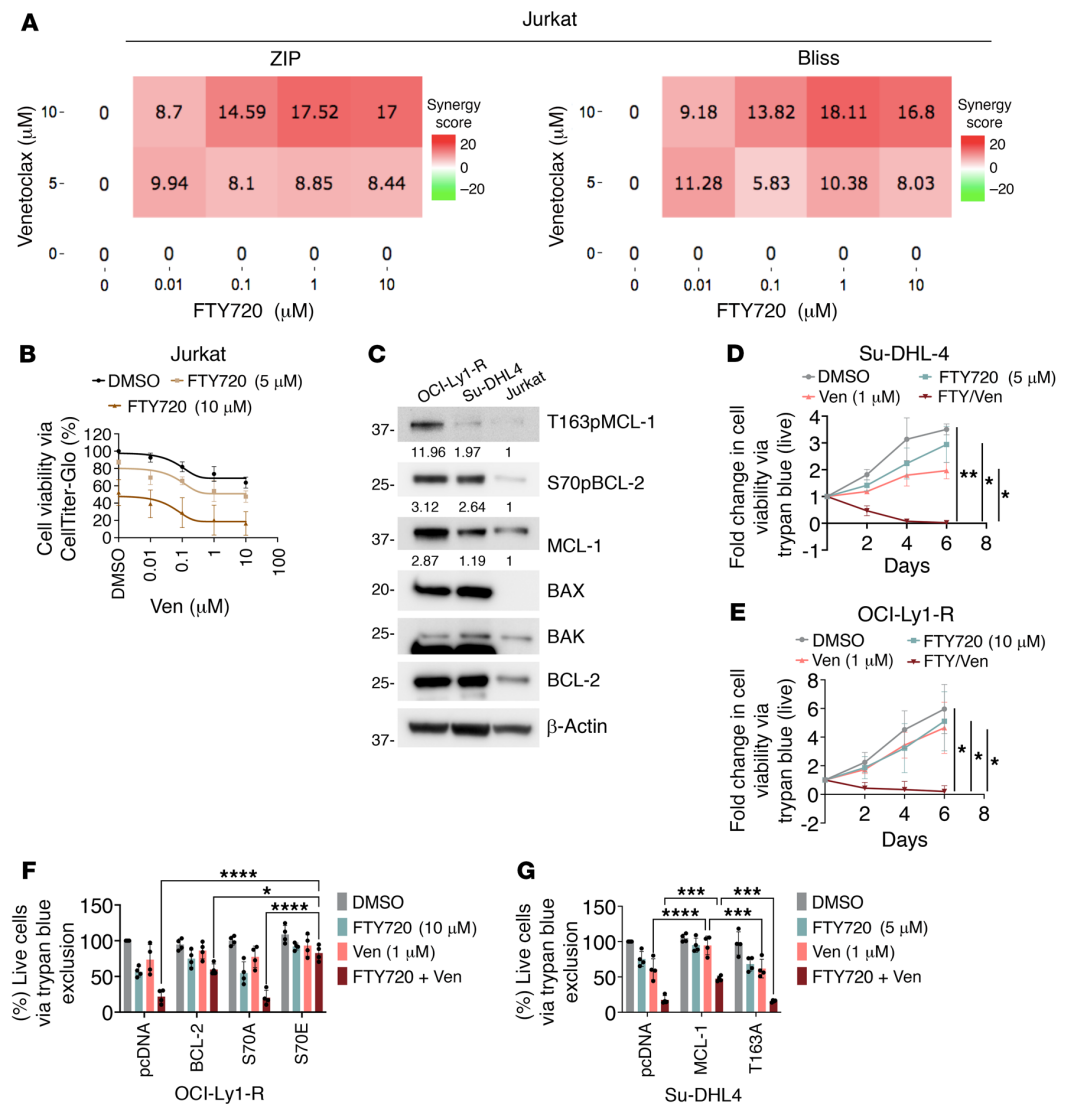
Rewiring to BCL-2 dependence and resensitization to venetoclax-induced cell death require a reduction of BCL-2 family protein phosphorylation. (**A**) Synergy scores for Jurkat were computed by SynergyFinder based on ZIP and Bliss Synergy models. Red indicates synergism and green indicates antagonism. (**B**) Viability of Jurkat (*n* = 3) cells following a 4-hour pretreatment with increasing concentrations of FTY720 and subsequent 48 hours of increasing concentrations of cotreatment with ABT199/venetoclax, measured by CTG assay. (**C**) Western blots showing the different levels of T163pMCL-1, S70pBCL-2, MCL-1, BAX, BAK, and BCL-2 in OCI-Ly1-R, Su-DHL4, and Jurkat cells. (**D** and **E**) Viability of live Su-DHL4 (*n* = 3) and OCI-Ly1-R (*n* = 4) cells following a 4-hour pretreatment with FTY720 and a subsequent cotreatment with ABT199/venetoclax at a time-chase of 2, 4, and 6 days, measured by trypan blue exclusion assay. The drug concentrations used were based on the highest/best synergy score. Tukey’s multiple-comparison test was used to determine significance. (**F**) Viability of live pcDNA3.1, WT BCL-2, p.S70A, and p.S70E transiently transfected OCI-Ly1-R cells following pretreatment with FTY720 (10 μM) for 4 hours and a subsequent cotreatment with ABT199/venetoclax (1 μM) for 48 hours, measured by trypan blue exclusion assay (*n* = 4). Dunnett’s multiple-comparison test was used. (**G**) Viability of live pcDNA3.1, WT MCL-1, and p.T163A transiently transfected Su-DHL4 cells following pretreatment with FTY720 (5 μM) for 4 hours and a subsequent cotreatment with ABT199/venetoclax (1 μM) for 24 hours, measured by trypan blue exclusion assay (*n* = 4). Dunnett’s multiple-comparison test was used. *P < 0.05, **P < 0.01, ***P < 0.001, and ****P < 0.0001.

Given that venetoclax-resistant cells were now BCL-2 dependent following FTY720 treatment, we hypothesized that the FTY720 and venetoclax combination would kill the previously resistant cells. We first pretreated resistant cells with FTY720 for 4 hours (to rewire cells toward BCL-2 dependence) and subsequently cotreated them with venetoclax for 24 or 48 hours. Indeed, we observed that both intrinsic and acquired-resistance lymphoid lines were sensitized to venetoclax following FTY720 pretreatment (Figure 8, A and B). In an unbiased manner, synergy score heatmaps were computed using 4 different models, including ZIP, HSA, Loewe, and Bliss, all of which demonstrated that the combination of FTY720 plus venetoclax was synergistic in both intrinsic and acquired resistance lines (Figure 8, C and D, and Supplemental Figure 4, A and B). We further demonstrated that this synergism requires the presence of phosphorylated BCL-2 family proteins, as relatively lower-to-undetectable S70pBCL-2, T163pMCL-1, and BAX expression in Jurkat cells displayed a more additive cytotoxic effect, as shown by 2 (ZIP and Bliss) synergy models and cell death curves when treated with the drug combination (Figure 9, A–C, and Supplemental Figure 4C). Further supporting this observation, FTY720 did not further enhance venetoclax-induced cell death in OCI-Ly1-S cells (Supplemental Figure 5A), which had lower basal levels of BCL-2 family protein phosphorylation (Figure 1B).

To demonstrate that this combination is cytotoxic (rather than cytostatic), we chased this treatment combination for 6 days with a trypan blue exclusion assay using the drug combination doses with the best synergy scores and found that the combination effectively reduced live cell counts of Su-DHL4 and OCI-Ly1-R (Figure 9, D and E), while reciprocally increasing their dead cell counts (Supplemental Figure 5, B and C), thus indicating that this treatment combination was indeed cytotoxic. These observations were further corroborated by an annexin-V/Hoechst assay (Supplemental Figure 5, D and E).

To further confirm that the cytotoxic effects of this combination require the reduction of BCL-2 family protein phosphorylation, we demonstrated that p.S70E, but not p.S70A, transfection of OCI-Ly1-R cells and WT MCL-1, but not p.T163A, transfection of Su-DHL4 cells resulted in resistance to the treatment combination, as measured by trypan blue exclusion and CellTiter-Glo (CTG) assays (Figure 9, F and G, and Supplemental Figure 5, F and G). Collectively, our findings indicate that FTY720 reprogrammed the kinome enzymatic landscape, reduced the levels of phosphorylation in BCL-2 family proteins, and rewired the apoptotic interactome and venetoclax-resistant lymphoid cell dependence from MCL-1 to BCL-2, thus resensitizes them to venetoclax-induced killing.

### Targeting hyperphosphorylated BCL-2 family proteins involves PP2A.

**Figure 10:**
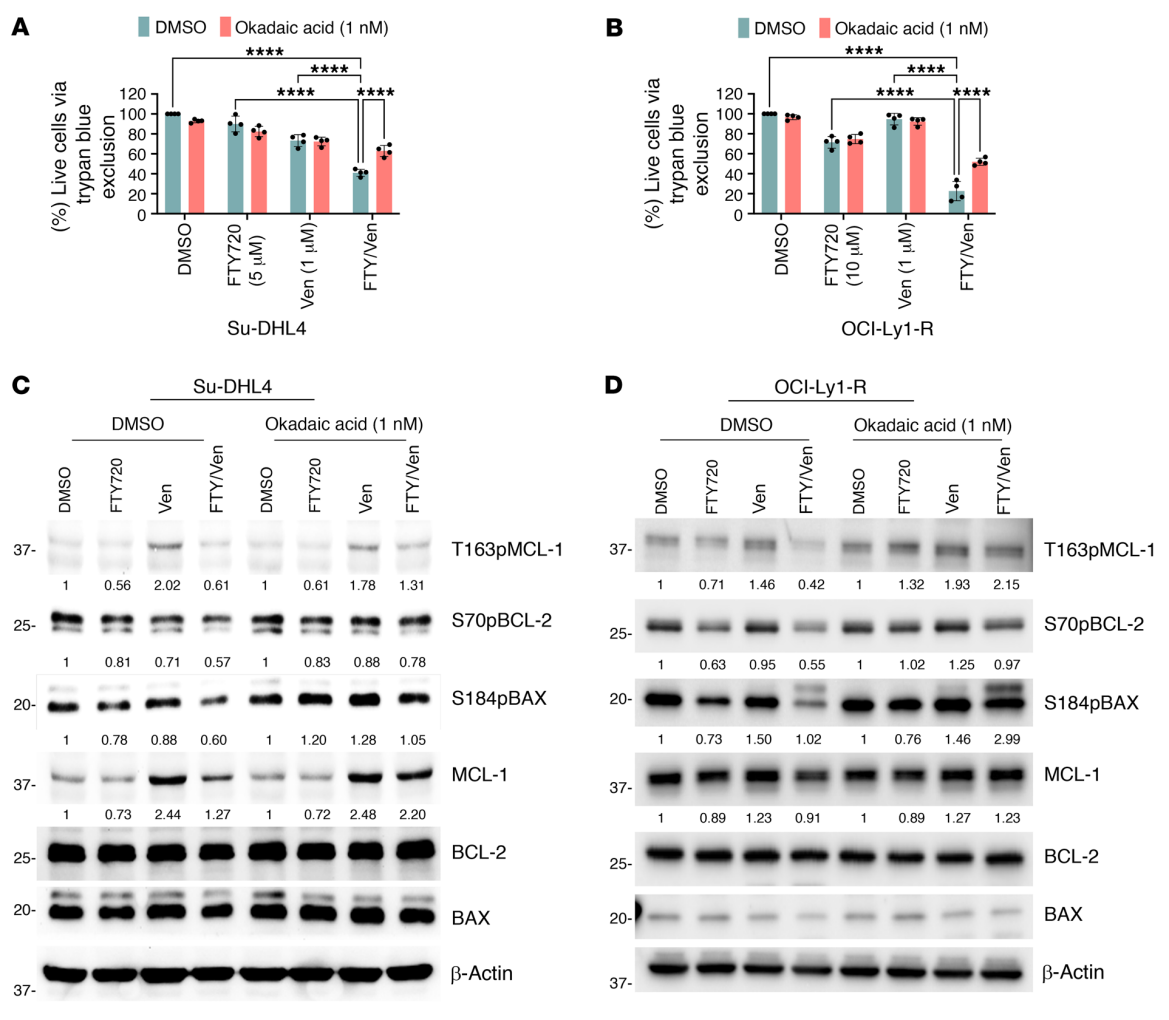
Reduced BCL-2 family protein phosphorylation is a function of PP2A. (**A** and **B**) Viability of Su-DHL4 and OCI-Ly1-R cells following pretreatment with OA (1 nM) for 2 hours followed by FTY720 (10 μM) for 4 hours and ABT199/venetoclax (1 μM) for 48 hours cotreatments, measured by trypan blue exclusion assay. (*n* = 4). Šidák’s multiple-comparison tests was used. ****P < 0.0001. (**C** and **D**) Western blots showing reversal of T163pMCL-1, S70pBCL-2, S184pBAX, and MCL-1 reductions in Su-DHL4 or OCI-Ly1-R cells following pretreatment with OA (1 nM) for 2 hours followed by FTY720 (10 μM) for 4 hours and ABT199/venetoclax (1 μM) for 24 hours of cotreatment.

Since FTY720 is a PAD (42) and hyperphosphorylated BCL-2 family proteins were all decreased by FTY720, we next sought to assess whether the effects of FTY720 were indeed related to PP2A activation. We pretreated Su-DHL4 or OCI-Ly1-R cells with okadaic acid (OA), a PP2A inhibitor, or an siRNA of the PP2A catalytic (siPP2Ac) subunit prior to treatment with FTY720 and venetoclax. Pretreatment with OA or siPP2Ac modestly reduced the cytotoxic effect of FTY720 and venetoclax combination (Figure 10, A and B, and Supplemental Figure 6, A–E). We also observed that OA or siPP2Ac was able to prevent the reduction of S70pBCL-2, T163pMCL-1, MCL-1, and S184pBAX levels by FTY720 (Figure 10, C and D, and Supplemental Figure 6F). To further validate these findings, we demonstrated that a different PAD, perphenazine (PPZ) (43), showed a similar effect in resensitizing venetoclax-resistant cells (Supplemental Figure 6G).

**Figure 11:**
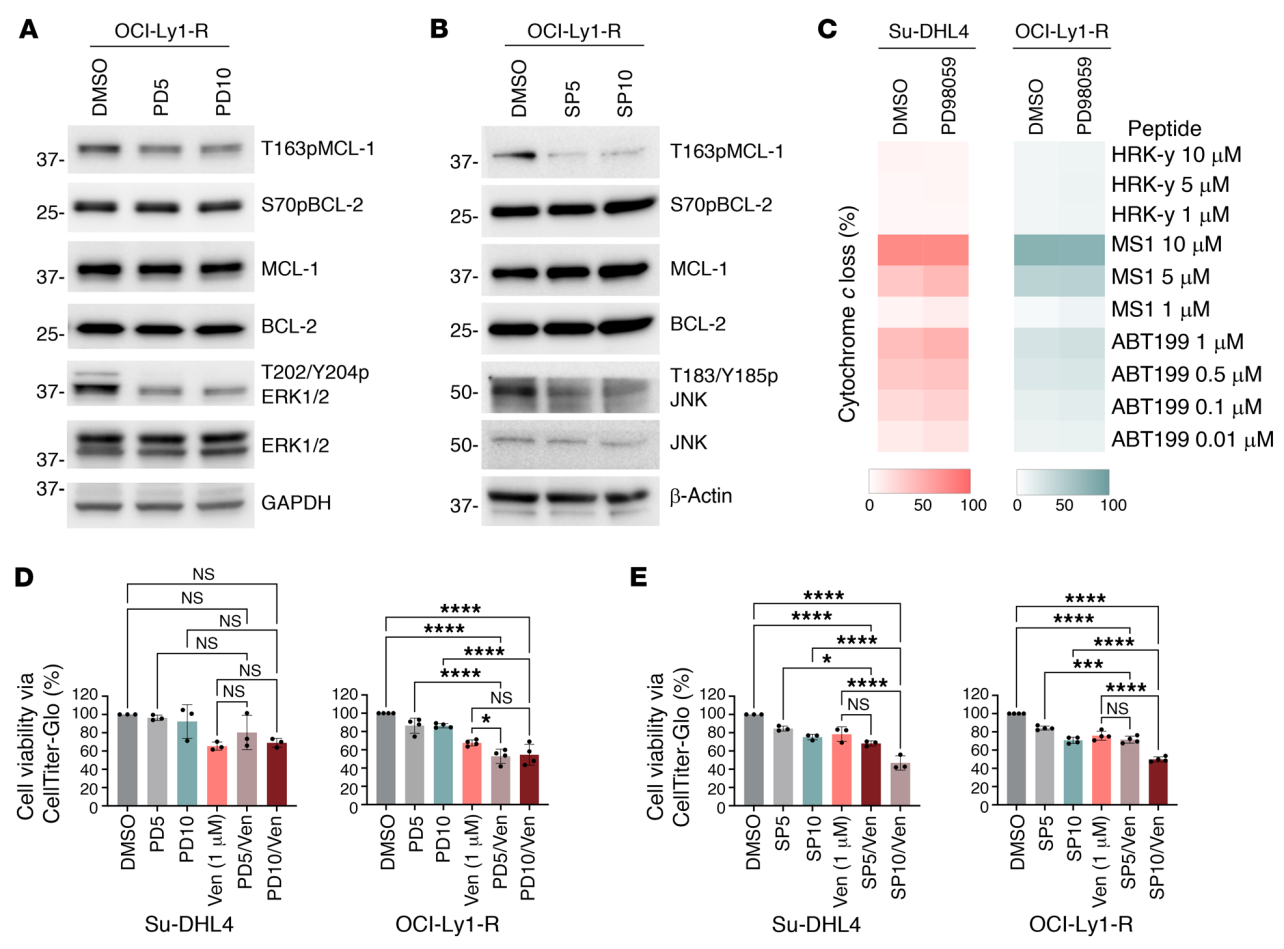
Inhibiting kinases singly fails to resensitize resistant cells to venetoclax. (**A**) Western blots showing unapparent changes in T163pMCL-1, S70pBCL-2, and MCL-1 levels in OCI-Ly1-R cells following treatment with increasing concentrations of the MEK/ERK1/2 inhibitor PD98059 (5 μM and 10 μM) for 24 hours. A reduction in T202/Y204pERK1/2 was used as a positive control. (**B**) Western blots showing unapparent changes of T163pMCL-1, S70pBCL-2, and MCL-1 in OCI-Ly1-R cells following increasing treatment concentrations of the JNK inhibitor SP600125 (5 μM and 10 μM) for 24 hours. A reduction in T183/Y185pJNK was used as a positive control. (**C**) DBP of Su-DHL4 and OCI-Ly1-R cells following treatment with PD98059 (10 μM) for 4 hours (*n* = 4). (**D**) Viability of Su-DHL4 (*n* = 3) and OCI-Ly1-R (*n* = 4) cells following pretreatment with PD98059 (5–10 μM) for 4 hours and subsequent cotreatment with ABT199/venetoclax (1 μM) for 48 hours, as measured by CTG assay. Šidák’s multiple-comparison test was used. (**E**) Viability of Su-DHL4 (*n* = 3) and OCI-Ly1-R (*n* = 4) cells following pretreatment with SP600125 (5–10 μM) for 4 hours and cotreatment with ABT199/venetoclax (1 μM) for 48 hours, as measured by CTG assay. Šidák’s multiple-comparison test was used. *P < 0.05, ****P < 0.0001.

As a negative control to demonstrate that these effects were PP2A specific and involved FTY720-induced kinome enzymatic reprogramming, we showed that exposure of OCI-Ly1-R or Su-DHL4 cells to a single kinase inhibitor of MEK/ERK1/2 (PD98059) or JNK (SP600125) failed to reduce S70pBCL-2, T163pMCL1, and MCL-1, rewire cellular dependence to BCL-2, or resensitize cells to venetoclax to the degree observed with FTY720 (Figure 11, A–E, and Supplemental Figure 7, A and B). These effects were intensified when PD98059 and SP600125 were used simultaneously to inhibit more than 1 kinase (Supplemental Figure 7, C–E). These findings therefore support the involvement of an altered enzymatic kinome, PP2A, and the use of PADs in resensitizing resistant cells to venetoclax.

### Targeting phosphorylation of BCL-2 family proteins sensitizes primary cells from treatment-naive CLL patients to venetoclax.

**Figure 12:**
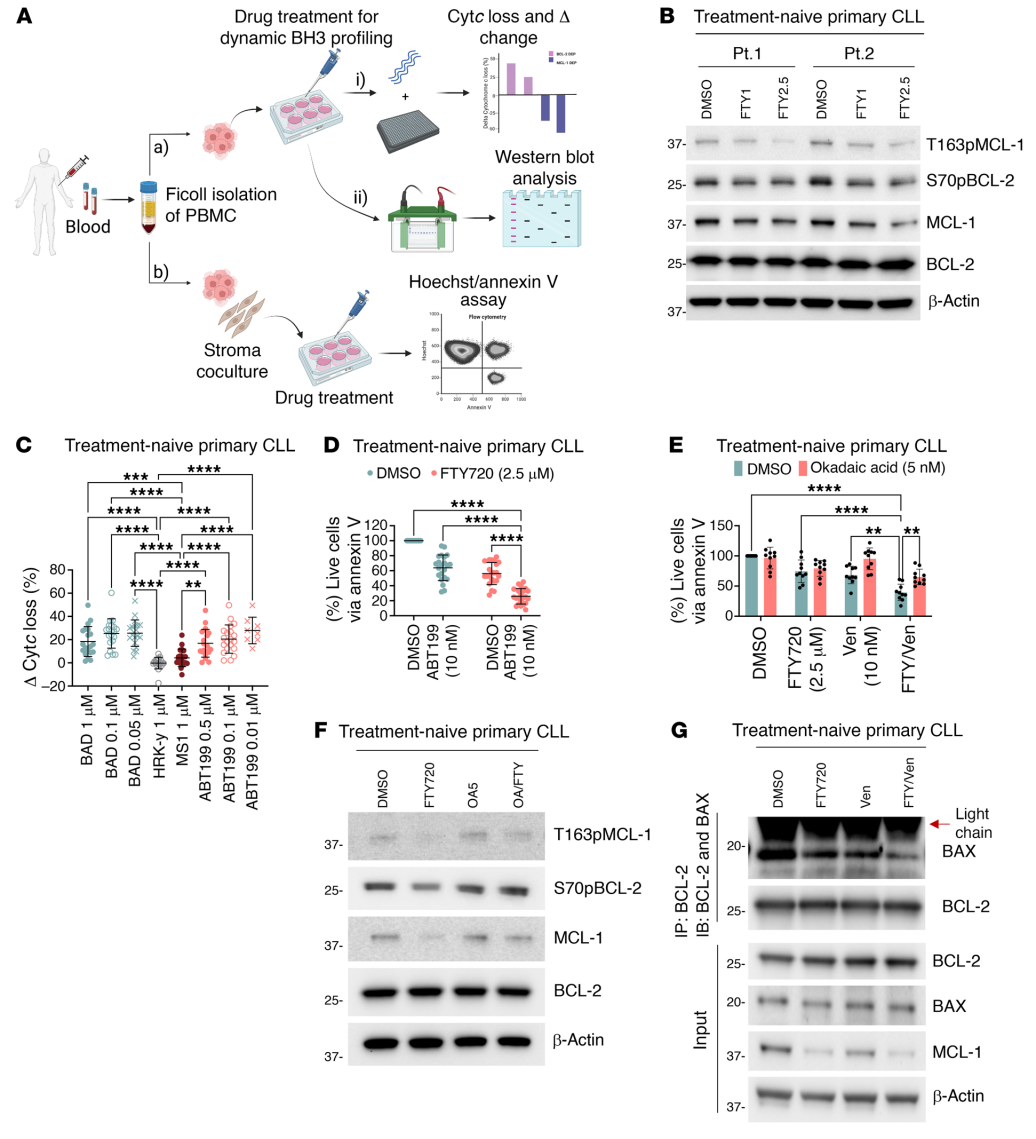
FTY720-induced PP2A activation displays similar cytotoxic effects on treatment-naive primary CLL cells. (**A**) Ficoll isolation of PBMCs from 28 treatment-naive patients with CLL. Samples were divided for DBP and Western blotting following ex vivo FTY720 treatment or were cocultured with NKTert stromal cells for cell viability measurement following FTY720 and ABT199/venetoclax cotreatments. The number of experiments performed was based on patient sample availability. The diagram was generated using BioRender software. (**B**) Western blots showing decreased T163pMCL-1 (*n* = 12), S70pBCL-2 (*n* = 18), and MCL-1 (*n* = 18) in treatment-naive primary CLL cells following increasing treatment concentrations of FY720 (1μM and 2.5 μM) for 4 hours. S112pBAD and S184pBAX were not evaluated because of insufficient samples. Quantification is displayed in Supplemental Figure 8A. (**C**) Δ Changes in the percentage of Cyt*c* loss between ex vivo FTY720 (2.5 μM) and DMSO treatments for 4 hours in treatment-naive primary CLL cells (*n* = 20) indicated increased BCL-2 dependence. Šidák’s multiple-comparison test was used. (**D**) Viability of treatment-naive primary CLL cells following an ex vivo 4-hour pretreatment with 2.5 μM (*n* = 22) FTY720 and a subsequent 24-hour cotreatment with 10 nM venetoclax, as measured by annexin V/Hoechst assay. Šidák’s multiple-comparison test was used. (**E**) Viability of treatment-naive primary CLL cells (*n* = 10) following ex vivo pretreatment with OA (5 nM) for 2 hours followed by FTY720 (2.5 μM) for 4 hours and ABT199/venetoclax (10 nM) for 48 hours of cotreatment, as measured by annexin V/Hoechst assay. Šidák’s multiple-comparison test was used. (**F**) Western blots showing reversal of T163pMCL-1, S70pBCL-2, and MCL-1 reductions in treatment-naive primary CLL cells (*n* = 8) following ex vivo pretreatment with OA (5 nM) for 2 hours followed by FTY720 (2.5 μM) cotreatment for 4 hours. Quantification is displayed in Supplemental Figure 10A. (**G**) Co-IP of BCL-2 and immunoblotting of BAX and BCL-2 in treatment-naive primary CLL cells (*n* = 3) following ex vivo 4-hour pretreatment with FTY720 (2.5 μM) and 1-hour cotreatment with ABT199/venetoclax (10 nM). Quantification is displayed in Supplemental Figure 10B. **P < 0.01, ***P < 0.001, and ****P < 0.0001.

To assess whether our cell line findings could be recapitulated in primary CLL cells from treatment-naive patients, we used PBMCs from 28 patients with CLL with various disease characteristics (Supplemental Table 1), treated the cells ex vivo with OA, FTY720, and/or venetoclax, and performed DBP, Western blotting, cell viability testing, and co-IP (Figure 12A). Similar to what we observed in the in vitro setting, S70pBCL-2, T163pMCL-1, and MCL-1 levels were reduced in CLL patient samples treated ex vivo with FTY720 (Figure 12B and Supplemental Figure 8A). S112pBAD and S184pBAX were omitted because of an insufficient amount of primary samples from patients. FTY720 also increased BCL-2 dependence in these treatment-naive primary CLL cells (Figure 12C), which subsequently increased sensitivity to venetoclax (Figure 12D). This cytotoxic effect was observed at concentrations as low as 1 μM FTY720 (Supplemental Figure 9A), a clinically achievable concentration. Furthermore, combination treatment with FTY720 (1 μM) and venetoclax minimally affected the viability of normal cells (PBMCs, T cells, and B cells) from healthy donors compared with that of primary CLL cells (Supplemental Figure 9, A–D). This further supports the feasibility of exploring this combination in the clinic. We again confirmed that these effects of FTY720 were related to PP2A, as OA could reverse the cytotoxic effects of the treatment combination and prevent the reduction of S70pBCL-2, T163pMCL-1, and MCL-1 levels (Figure 12, E and F, and Supplemental Figure 10A). Additionally, we observed that the combination of FTY720 and venetoclax enhanced the dissociation of BAX from BCL-2 (Figure 12G and Supplemental Figure 10B). Collectively, these ex vivo results from treatment-naive primary CLL samples were highly consistent with our cell line findings, suggesting that the mechanisms we have elucidated were likely relevant to the biology of CLL in patients.

### Comparison of paired samples from CLL patients at pretreatment to progression while on/off venetoclax supports the potential utility of PADs in enhancing sensitivity to venetoclax.

**Figure 13:**
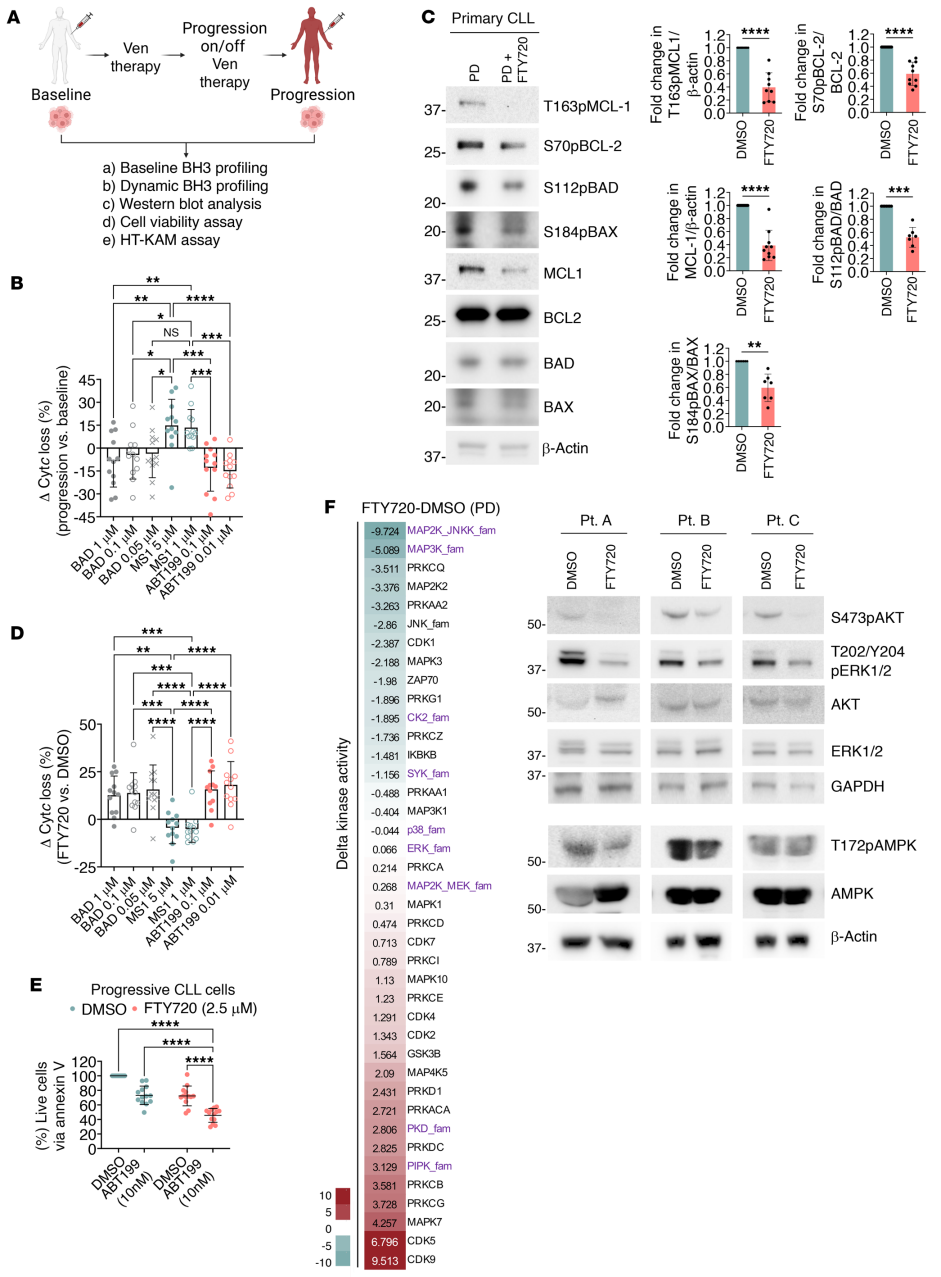
Hyperphosphorylation of BCL-2 family protein–driven venetoclax resistance in patients with progressive primary CLL could be targeted by PAD. (**A**) Thirteen paired pre-venetoclax and PD patient primary CLL cells were used for specified experiments. The number of experiments performed is based on patient sample availability. (**B**) Δ Changes of the percentage of Cyt*c* loss between paired PD and pre-venetoclax patient primary CLL cells (*n* = 12) indicated an increase in MCL-1 and a drop in BCL-2 dependencies. (mean + SD). Šidák’s comparisons test was used. (**C**) Western blots of PD samples treated ex vivo with FTY720 (2.5 μM) for 4 hours showed decreased T163pMCL-1 (*n* = 9), S70pBCL-2 (*n* = 10), MCL-1 (*n* = 10), S112pBAD (*n* = 7), and S184pBAX (*n* = 7). Paired *t* tests were used. (**D**) Δ Changes in the percentage of Cyt*c* loss between FTY720 (2.5 μM) and DMSO ex vivo treatments in PD primary CLL cells (*n* = 12) indicated an increase in BCL-2 and a drop in MCL-1 dependencies. Data represent the mean + SD. Šidák’s multiple-comparison test was used. (**E**) Viability of PD primary CLL (*n* = 13) cells following a 4-hour ex vivo pretreatment with FTY720 (2.5 μM) and a 24-hour cotreatment with venetoclax (10 nM), measured by annexin V/Hoechst assay. Šidák’s multiple-comparison test was used. (**F**) HT-KAM analyses of 2 PD primary CLL samples treated ex vivo with FTY720 (2.5 μM) for 4 hours. Western blots show decreased S473pAKT, T202/Y204pERK1/2, and T172pAMPK levels in PD primary CLL cells following ex vivo treatment with FTY720 (2.5 μM) for 4 hours. The same samples were run on 2 separate gels. *P < 0.05, **P < 0.01, ***P < 0.001, and ****P < 0.0001.

To further explore this functional mechanism of venetoclax resistance, we studied 13 paired samples from patients with CLL to compare pretreatment baseline with the time of progression while on venetoclax (Figure 13A and Supplemental Table 2). Compared with their paired pre-venetoclax samples, the progression samples had increased MCL-1 and reciprocally reduced BCL-2 dependencies (Figure 13B). This finding is consistent with the significant increases in T163pMCL-1, S70pBCL-2, S112pBAD, and MCL-1 observed in progression samples relative to their paired pre-venetoclax samples, as shown earlier (Supplemental Figure 1C and Figure 1C, samples from the same patients). We next treated these paired pre-venetoclax and progression CLL samples with venetoclax ex vivo and observed that the progression samples were indeed more resistant to venetoclax-induced cell death (Supplemental Figure 11A). These observations support the hypothesis that venetoclax resistance in lymphoid malignancies is due at least in part to hyperphosphorylated BCL-2 family proteins, which leads to a switch from BCL-2 dependence to MCL-1 dependence.

We next tested whether ex vivo treatment with FTY720 could resensitize these progression samples to venetoclax. Indeed, we found that FTY720 simultaneously reduced T163pMCL-1, S70pBCL-2, S112pBAD, S184pBAX, and MCL-1 levels (Figure 13C), rewired dependence from MCL-1 to BCL-2 (Figure 13D), and thereby resensitized venetoclax-resistant primary CLL cells to venetoclax (Figure 13E). To explore whether these resistant primary samples would also undergo kinome enzymatic reprogramming following FTY720 treatment, like we observed in the cell lines (Figure 4E), we examined the kinome enzymatic activity of 2 venetoclax progression samples from patients with CLL following ex vivo FTY720 treatment using the HT-KAM assay. Indeed, we observed an altered kinome landscape in these samples from patients with CLL whose disease was progressing on venetoclax, in which many of the suppressed kinases were from the MAPK family that affects the phosphorylation of BCL-2, MCL-1, BAD, and BAX (Figure 13F). We further confirmed the suppression of the activity of several kinases by showing reduced phospho-activation sites of AMPK, AKT, and ERK via Western blotting (Figure 13F).

### A venetoclax-resistant aggressive B cell lymphoma mouse model further supports the ability of PADs to resensitize venetoclax resistance in vivo.

**Figure 14:**
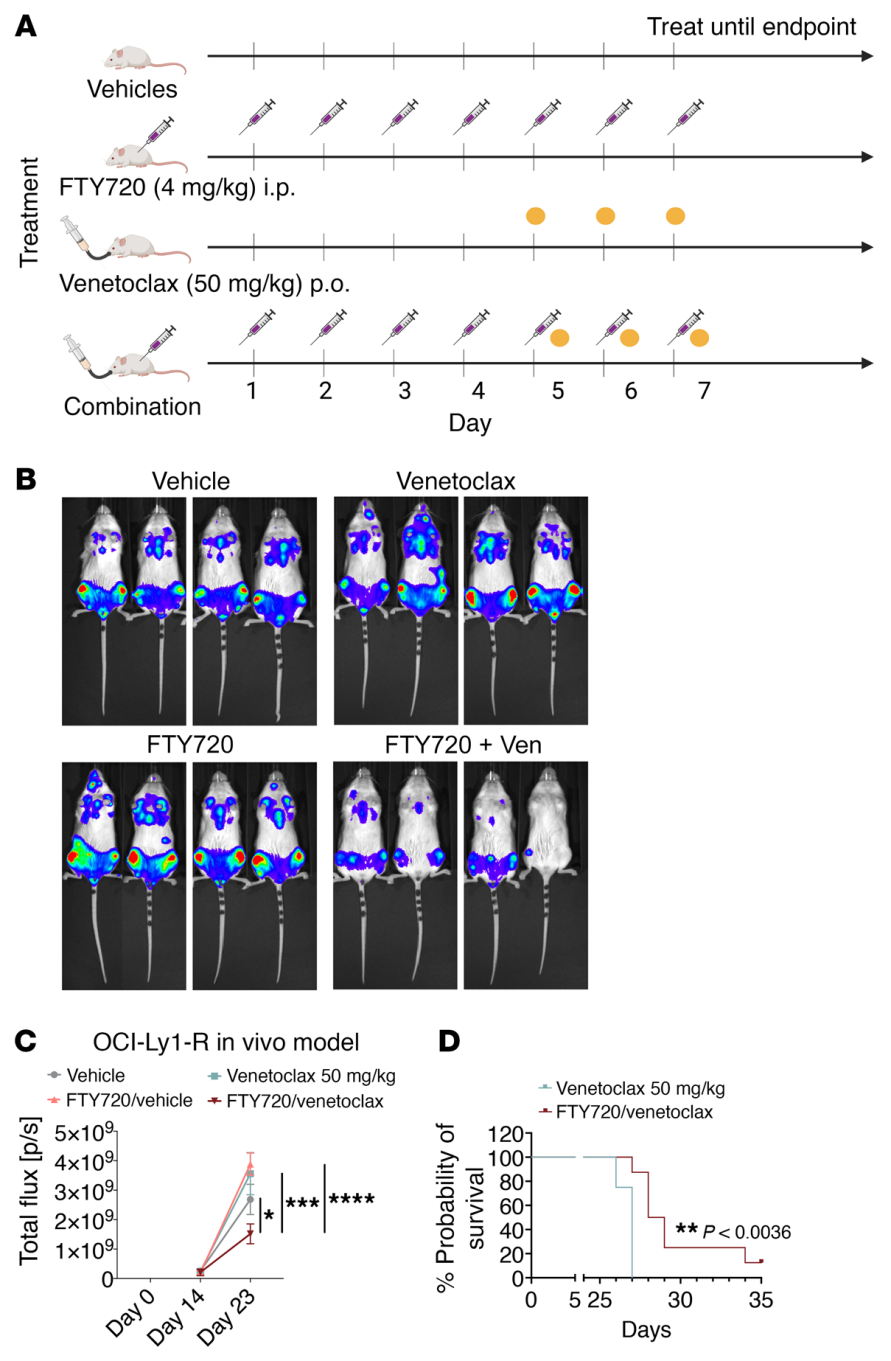
Sequential treatment combination with FTY720 and venetoclax reduces tumor burden and prolongs the survival of mice in an OCI-Ly1-R–resistant murine model. (**A**) FTY720 and venetoclax treatment schedule for NSG mice implanted with luciferase-tagged OCI-Ly1-R cells via tail-vein injection. FTY720 (4 mg/kg/day, i.p.) was administered from days 1–4 and subsequently in combination with ABT199/venetoclax (50 mg/kg/day, p.o.). The diagram was generated with BioRender software. (**B**) Representation of BLI of luciferase-tagged OCI-Ly1-R–implanted NSG mice subjected to the respective treatments at day 23. (**C**) Quantified total flux (p/s) of BLI (mean ± SEM) for luciferase-tagged OCI-Ly1-R–implanted NSG mice subjected to the respective treatments: vehicle (*n* = 6), venetoclax (*n* = 4), FTY720 (*n* = 8), or FTY720 plus venetoclax (*n* = 8). Dunnett’s multiple-comparison test was used. (**D**) Survival percentage probability for luciferase-tagged OCI-Ly1-R–implanted NSG mice treated with venetoclax or FTY720 plus venetoclax. The Gehan-Breslow-Wilcoxon test was used. *P < 0.05, **P < 0.01, ***P < 0.001, and ****P < 0.0001.

We used an acquired venetoclax-resistant OCI-Ly1-R murine model to evaluate whether FTY720 could overcome venetoclax resistance in vivo. A stable, Luc-tagged OCI-Ly1-R cell line was implanted into NOD/SCID/Il2Rγ^–/–^ (NSG) mice via tail-vein injection, and the mice were randomized and subjected to bioluminescence imaging (BLI) for luciferase signals on day 14. Mice without tumors were removed, while the rest were then subjected to specific treatment schedules on day 16 (Figure 14A). BLI on day 23 showed that, whereas single-agent venetoclax or FTY720 failed to induce any appreciable therapeutic effect, the combination of the 2 agents resulted in a significantly lower tumor burden (Figure 14, B and C). The resistance of OCI-Ly1-R murine model to venetoclax was also verified in vivo when compared with the sensitive OCI-Ly1-S murine model (Supplemental Figure 11B). Importantly, mice harboring venetoclax-resistant cells had a significantly longer survival in the combination arm compared with controls, particularly for mice in the venetoclax monotherapy arm (Figure 14D and Supplemental Figure 11, C and D). These in vivo data corroborate our in vitro and ex vivo data and collectively suggest that FTY720 has the potential to overcome venetoclax resistance in patients with lymphoid malignancies.

## Discussion

We have uncovered a functional mechanism of venetoclax resistance in lymphoid malignancies driven by hyperphosphorylation of the BCL-2 family proteins BCL-2, MCL-1, BAD, and BAX. We demonstrate that heightened BCL-2 family protein phosphorylation modulated the apoptotic protein interactome and altered tumor cell survival dependence from BCL-2 to MCL-1, thus diminishing the sensitivity of malignant lymphoid cells to venetoclax. Importantly, we demonstrate an approach that addresses this resistance mechanism through the use of PADs to reprogram the resistance-state kinome enzymatic activity and dephosphorylate BCL-2 family proteins, thereby rewiring the apoptotic interactome and survival dependence back to BCL-2 and resensitizing resistant cells to venetoclax. The consistency of our results across paired sensitive and resistant cell lines, an in vivo mouse model, and in primary samples from patients with CLL speaks to the robustness of these findings.

Our findings build on the results of our prior work that first detected elevated levels of AMPK and MCL-1 in venetoclax-resistant lymphoid malignant cells (44). We believe our new data are particularly noteworthy because AMPK has been demonstrated to positively regulate the phosphorylation and expression of BCL-2 and MCL-1, respectively (36, 37, 45–47). Further supporting the involvement of AMPK are our HT-KAM results showing enhanced AMPK activity (PRKAA1/2) in resistant cells, which was abrogated in the presence of FTY720 (Figure 4E). Additionally, as AKT and ERK also phosphorylate and inactivate BAX (S184pBAX) and BAD (S112pBAD), whereas GSK3β phosphorylates and destabilizes MCL-1 (S159pMCL-1) (17, 38, 40), our findings from Western blotting and HT-KAM assays demonstrating the inactivation of AKT and ERK as well as activation of GSK3β further corroborate the downstream changes in S184pBAX, S112pBAD, and S159pMCL-1. A detailed analysis of the mechanisms underlying the drastic change in the kinome enzymatic activity landscape for venetoclax resistance is beyond the scope of this work. But given that venetoclax resistance has previously been shown to increase ROS, and because ROS could regulate the enzymatic activity of kinases and S70pBCL-2 levels (11, 21, 44, 48), it is possible that ROS are a key upstream contributor to the drastic change in the kinome enzymatic landscape and BCL-2 family protein phosphorylation, leading to venetoclax resistance.

While genomic mechanisms including *BCL2* mutations have a critical role in some cases of venetoclax resistance, these mutations were often detected at low VAF in patients with resistance to venetoclax (4, 5). Moreover, BCL-2 inhibitors that could overcome such genomic resistance mechanisms have not yet emerged. Hence, it is imperative to minimize the occurrence of such mutations by efficiently and thoroughly eliminating tumor cells in a time-limited venetoclax therapy approach. One such approach being utilized in CLL is time-limited venetoclax plus anti-CD20 monoclonal antibody therapy, which could potentially reduce the risk of acquisition of *BCL2* mutations by minimizing the duration of venetoclax exposure (49). This approach therefore provides a rationale for the use of venetoclax plus PADs as another time-limited therapeutic option. Nonetheless, the functional resistance mechanism we have identified here may provide more straightforward therapeutic opportunities for patients with relapsed/refractory disease while on venetoclax due to the “druggability” of S70pBCL-2, T163pMCL-1, S112pBAD, and S184pBAX with several promising drug candidates such as PADs. Additionally, it is likely that functional and genomic resistance mechanisms are not mutually exclusive, and our data do not exclude the possibility that *BCL2* mutations could exist concomitantly with hyperphosphorylation of BCL-2 family proteins. An important remaining question is whether *BCL2* mutations are related in some way to BCL-2 phosphorylation (S70pBCL-2) at the BCL-2 flexible loop, or whether the co-occurrence of S70pBCL-2 and *BCL2* mutation(s) may further distort the conformation of BCL-2, leading to a further decrease in venetoclax binding.

Our data also suggest that venetoclax-resistant lymphoid cells may be sensitive to MCL-1 inhibition, as the resistant cells in our experiments generally depended on MCL-1 for survival. Dual targeting of both MCL-1 and BCL-2 could, in theory, overcome such a resistance mechanism; however, the development of direct MCL-1 inhibitors in the clinic has been limited by cardiotoxicity. Our findings here suggest that an alternative approach to MCL-1 inhibition that may have a better safety profile is to use PADs. In particular, we found that FTY720, a fingolimod compound that is FDA approved to treat multiple sclerosis, is a promising candidate to be studied in the clinic as a partner for venetoclax. Moreover, venetoclax is also being studied in solid tumors. For example, in breast cancer, in which BCL-2 family protein phosphorylation is implicated in tumor progression and drug resistance (50–52), the resistance mechanism of hyperphosphorylation of BCL-2 family proteins we identified here may provide a shared insight into venetoclax resistance in this disease. The addition of PADs could therefore also be explored in this context.

The significant changes we observed in the kinome enzymatic activity of venetoclax-resistant cells represent another important finding from our HT-KAM assay that supports the use of PADs. Our data suggest that targeting only 1 kinase, such as ERK1/2 or JNK, to overcome resistance may not be sufficient (Figure 11, A–E and Supplemental Figure 7, A and B). The lower cytotoxic activity of these combinations compared with that achieved with PADs could relate to the various redundant kinases that could sustain the phosphorylation of S70pBCL-2, T163pMCL-1, S112pBAD, and S184pBAX in a compensatory manner (7, 13–15, 36–40). Given that multiple individual kinases have recently been reported to affect the efficacy of venetoclax by different research groups, it is likely that venetoclax resistance relies on more than 1 hyperactivated kinase, as we found with the HT-KAM assay and simultaneous inhibition of JNK and ERK (Figure 4E and Supplemental Figure 7, C–E). This suggests that targeting 1 kinase at a time may only serve to inhibit cancer growth transiently, with subsequent tumor progression being related to a variety of other kinases. Inhibiting each of these kinases with individual drugs would be prohibitively challenging in the clinical setting, thus highlighting the therapeutic potential of PADs as a way to target multiple kinases using a single drug as a combination partner for venetoclax. Nonetheless, we do not dismiss the possibility of identifying combinations of kinase inhibitors with tolerable safety profiles, particularly with individual kinase inhibitors that are already in clinical use. HT-KAM will be an efficient way to functionally screen, identify, and evaluate the many possible combinations to determine which kinase inhibitors would be most promising to test preclinically and explore further in the clinic in combination with venetoclax. Collectively, our current findings support the further exploration of PADs and venetoclax in prospective clinical trials involving patients with lymphoid malignancies.

Finally, our work has the potential to open up new treatment approaches and to identify new therapeutic vulnerabilities for patients with lymphoid malignancies that are resistant to venetoclax. For example, the biological signatures we identified here allow the stratification of patients with lymphoid malignancies into different categories of resistance mechanisms, thus paving the way to more personalized therapeutic strategies (e.g., combination of PAD plus venetoclax or kinase inhibitors plus venetoclax in lymphoid malignancies expressing hyperphosphorylated BCL-2 family protein[s]). Coupled with the functional assays such as BH3-profiling and HT-KAM assays, our biological signatures could further assist in identifying lead compounds or repurposing FDA-approved drugs (e.g., FTY720) via compound/drug library screenings based on their potential to activate PP2A/inhibit kinases, reduce BCL-2 family protein phosphorylation, and rewire survival dependencies toward BCL-2 to facilitate venetoclax-induced cell death.

## Methods

Additional details on methods and materials can be found in the Supplemental Materials.

### Primary cells and cell lines.

PBMCs were isolated from whole blood obtained from consented patients with CLL using the Ficoll-Paque (GE Healthcare) density gradient centrifugation method. Isolated primary cells were either immediately used for experiments or viably frozen in freezing media (90% FBS, 10% DMSO) for subsequent experiments. The cell lines used in this study were OCI-Ly1, Su-DHL4, TOLEDO, TMD8, Jurkat, and Stroma-NKTert. The acquired-resistance cell lines OCI-Ly1-R and Su-DHL4-R were generated from parental cells following continuous culturing in venetoclax starting from a sublethal dose of 10 nM and then doubling this dose when cells could grow at a rate similar to that of its parental cells, up to 1 μM (44). Acquired venetoclax-resistant cells were previously tested through whole-exome sequencing and confirmed to not have *BCL2* mutations that have been reported to affect binding to venetoclax (4, 44). All primary cells and cell lines were cultured in RPMI-1640 medium supplemented with 10% FBS, 1% l-glutamine, 100 U/mL penicillin, and 100 μg/mL streptomycin. Routine mycoplasma testing was performed to ensure the absence of mycoplasma contamination.

### BH3-profiling technique.

BH3 profiling is a functional technique that measures the proximity of cells to apoptosis and the dependence of cells on specific antiapoptotic proteins for survival. Additional details are included in the Supplemental Methods.

### HT-KAM assay.

HT-KAM is a high-throughput functional platform that measures cellular kinase activity (57). Additional details are included in the Supplemental Methods.

### Cell viability.

Three different cell viability assays were used in this study: CTG, trypan blue exclusion, and annexin/Hoechst. The cell density used was approximately 0.75 million cells/mL. Additional details are included in the Supplemental Methods.

### Animal studies.

NOD/SCID/Il2Rγ^–/–^ (NSG) mice were purchased from The Jackson Laboratory. Luc-tagged OCI-Ly1-S or OCI-Ly1-R cells (1 million cells) were injected into the tail vein of 6- to 8-week-old female NSG mice, which were randomized to the treatment arms (*n* = 8 mice per arm). Treatments were administered until euthanization. BLI was performed for cell engraftment and tumor burden on mice using the Xenogen imager, and analysis was done using Living Image 4.2 software (PerkinElmer). Mice with no cell engraftment or tumor development were removed. Venetoclax was administered at 50 mg/kg/day p.o. (dissolved in 10% ethanol, 60% PHOSAL 50PG, and 30% PEG400), and FTY720 (dissolved in saline) was administered at 4 mg/kg/day i.p.

### Synergy analysis.

Synergy analyses for drug combinations were computed by SynergyFinder (https://synergyfinder.org/#!/) (58).

### Statistics.

Statistical analyses were performed using GraphPad Prism, version 9 (GraphPad Software). Data are presented as the mean ± SD (or as otherwise specified in the figure legends) of individual values, scatter plots, heatmaps, and bar graphs with triplicates or more for cell lines and single runs for primary samples. Statistical tests included a 2-tailed *t* test, 1-way ANOVA, 2-way ANOVA, and multiple-comparison tests, with statistical significance set at a *P* value of less than 0.05. Pearson’s correlation coefficient *R* was computed for correlations between 2 variables.

### Study approval.

Animal studies were performed under a protocol approved by the IACUC of the Dana-Farber Cancer Institute (DFCI). Patient samples were used with written informed consent and a protocol reviewed and approved by the Dana-Farber/Harvard Cancer Center IRB.

### Data availability.

All supporting data values are provided in the Supplemental Supporting Data Values file. Materials are available from the authors on reasonable request.

## Author contributions

SJFC and MSD conceptualized the study. SJFC, RG, CSM, JPC, and MSD designed the study methodology. SJFC, FZ, OD, RM, JXHL, LH, CER, MCC, JP, RC, YCH, MB, and DPM performed experiments. SJFC, FZ, OD, RM, JXHL, RC, YCH, MB, DPM, YCL, CSM, JPC, and MSD performed data analysis and interpretation. SJFC, LH, CER, MCC, JBI, CJW, SP, BCG, RRF, JNA, CSM, JPC, and MSD provided administrative, technical, material, or funding support. MSD supervised the study. SJFC, JPC, and MSD wrote the original draft of the manuscript. All authors reviewed and edited the manuscript.

## Supplementary Material

Supplemental data

Supporting data values

## Version 1 09/26/2023

In-Press Preview

## Version 2 11/15/2023

Electronic publication
