# Supplementary material for: Hyperphosphorylation of BCL-2 family proteins underlies functional resistance to venetoclax in lymphoid malignancies
Source: J Clin Invest. 2023 Nov 15;133(22):e170169. doi: 10.1172/JCI170169 (PMC10645378; doi:10.1172/JCI170169)
Supplement: Supplemental data [file jci-133-170169-s177.pdf]

Supplementary Figure 1

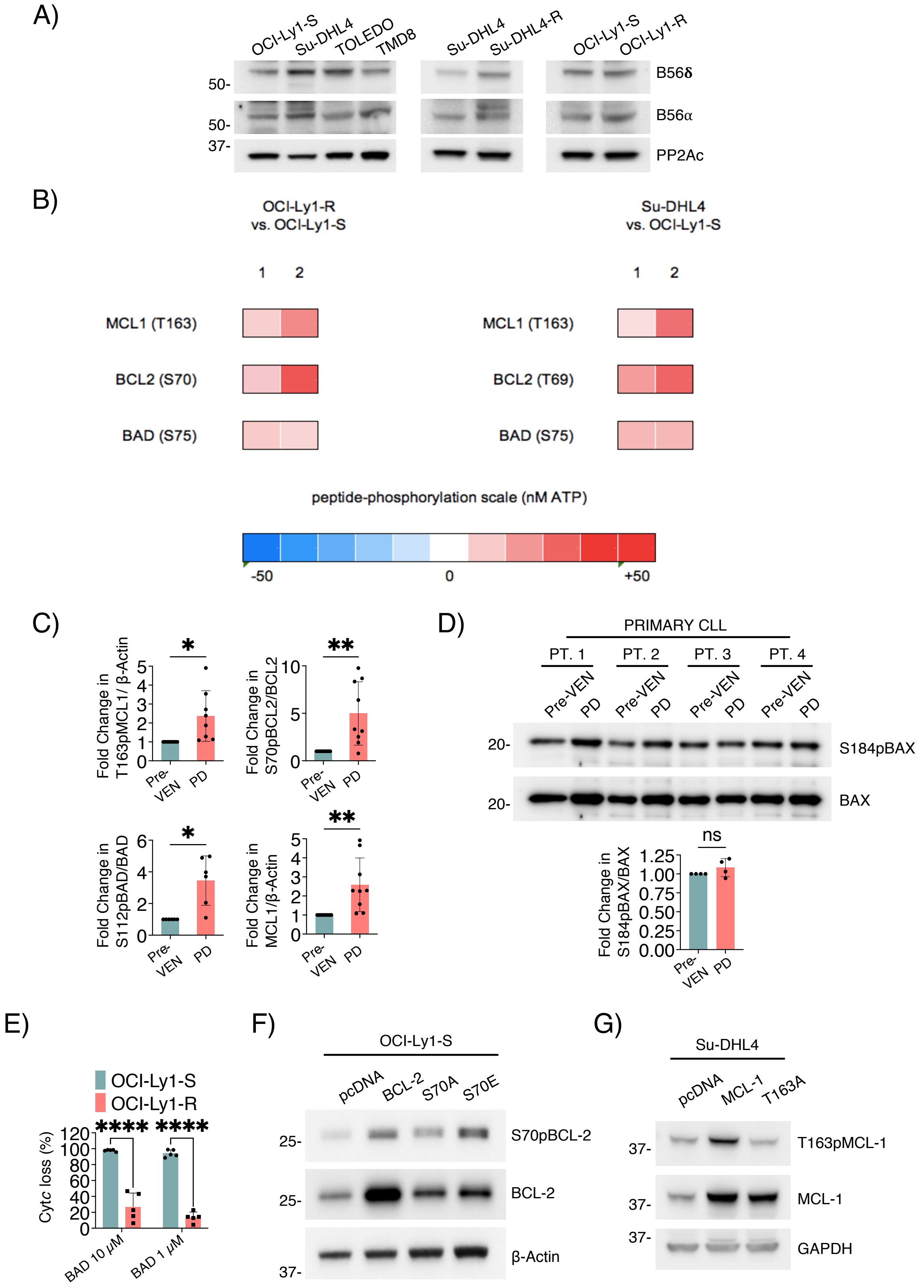

Supplemental Figure 1. Detection of BCL-2 family protein phosphorylation in resistant cells and BCL-2 and MCL-1 mutant-transfected cells.

A) Western blotting showing no substantial changes in PP2A subunit B56α, B45δ and PP2Ac expression in intrinsically and acquired resistant lymphoid malignant cells. These results are from the same sample sets run in figure 1B. Loading control is shown in figure 1B.

B) Δ change in phospho-peptide levels showing an increased T163pMCL-1, S70pBCL-2 and S75pBAD in OCI-Ly1-R or Su-DHL4 in comparison to OCI-Ly1-S, measured by HT-KAM assay. #1 and #2 indicate 2 replicates. S112pBAD nomenclature is based on mouse sequence. The analogous phosphorylation site of S112pBAD is S75pBAD in human. Information of S112pBAD antibody with the catalogue number #9291 could be found on Cell Signaling Technology.

C) Quantified normalized expression values derived from T163pMCL-1/β-Actin, S70pBCL-2/BCL-2, S112pBAD/BAD and MCL-1/β-Actin in progression (PD) primary CLL patient samples on venetoclax comparing to paired pre-venetoclax primary CLL patient samples for figure 1C. Bands quantified by ImageJ software. Paired t-test was used.

D) Western blot analysis showing no significant changes in S184pBAX in progression (PD) primary CLL patient samples on venetoclax as compared to paired pre-venetoclax primary CLL patient samples (human *in vivo*). (n=4). Bands quantified by ImageJ software. Paired t-test was used.

E) Baseline BH3-profiling of OCI-Ly1-R and OCI-Ly1-S following 1μM or 10μM BAD peptide incubation (n=5). Higher Cyt*c* release by BAD indicates higher BCL-2 dependence. Sidak's multiple comparison test was used.

F) Western blot analysis showing S70pBCL-2, BCL-2 and β-Actin levels following transient transfection with empty vector (pcDNA3.1), wild-type BCL-2, p.S70A and p.S70E in OCI-Ly1-S cells.

G) Western blot analysis showing T163pMCL-1, MCL-1 and β-Actin levels following transient transfection with pcDNA3.1, wild-type MCL-1 and p.T163A in Su-DHL4 cells.

*$p < 0.05$, **$p < 0.01$, ***$p < 0.001$, ****$p < 0.0001$ or otherwise stated.

Supplementary Figure 2

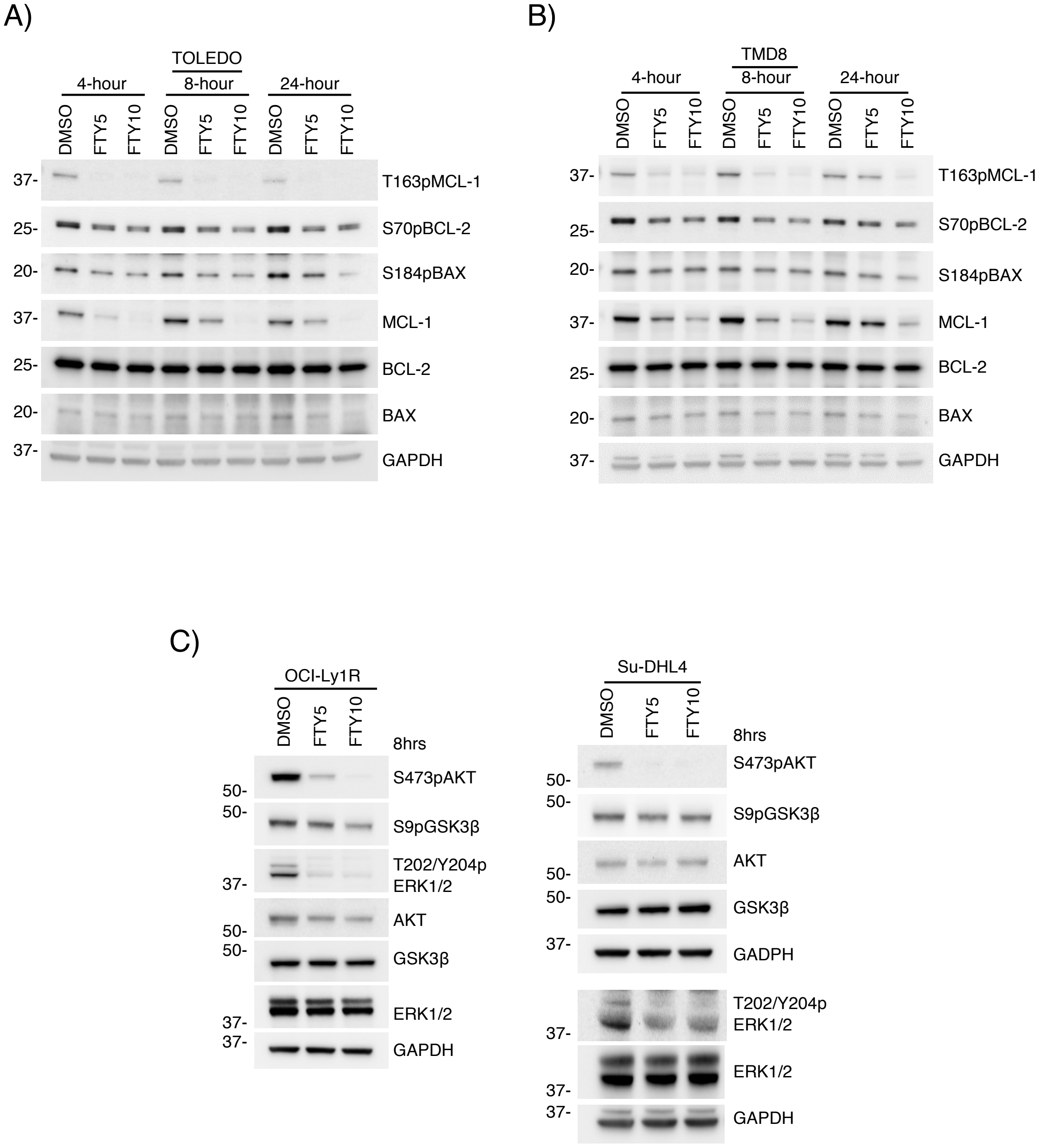

Supplemental Figure 2. FTY720 reduces phosphorylation levels of BCL-2 family proteins and multiple kinases.

A-B) Western blot analyses showing the reduction of T163pMCL-1, S70pBCL-2, S184pBAX, MCL-1 in TOLEDO and TMD8 cells following increasing treatment concentration (5-10μM) and incubation time of FTY720.

C) Western blot analyses showing the reduction of S473pAKT, S9pGSK3β and T202/Y204pERK1/2 in OCI-Ly1-R and Su-DHL4 cells following increasing treatment concentration of FTY720 (5-10μM) for 8 hours. Same sample for Su-DHL4 cells were run on 2 separate gels.

Supplemental Figure 3

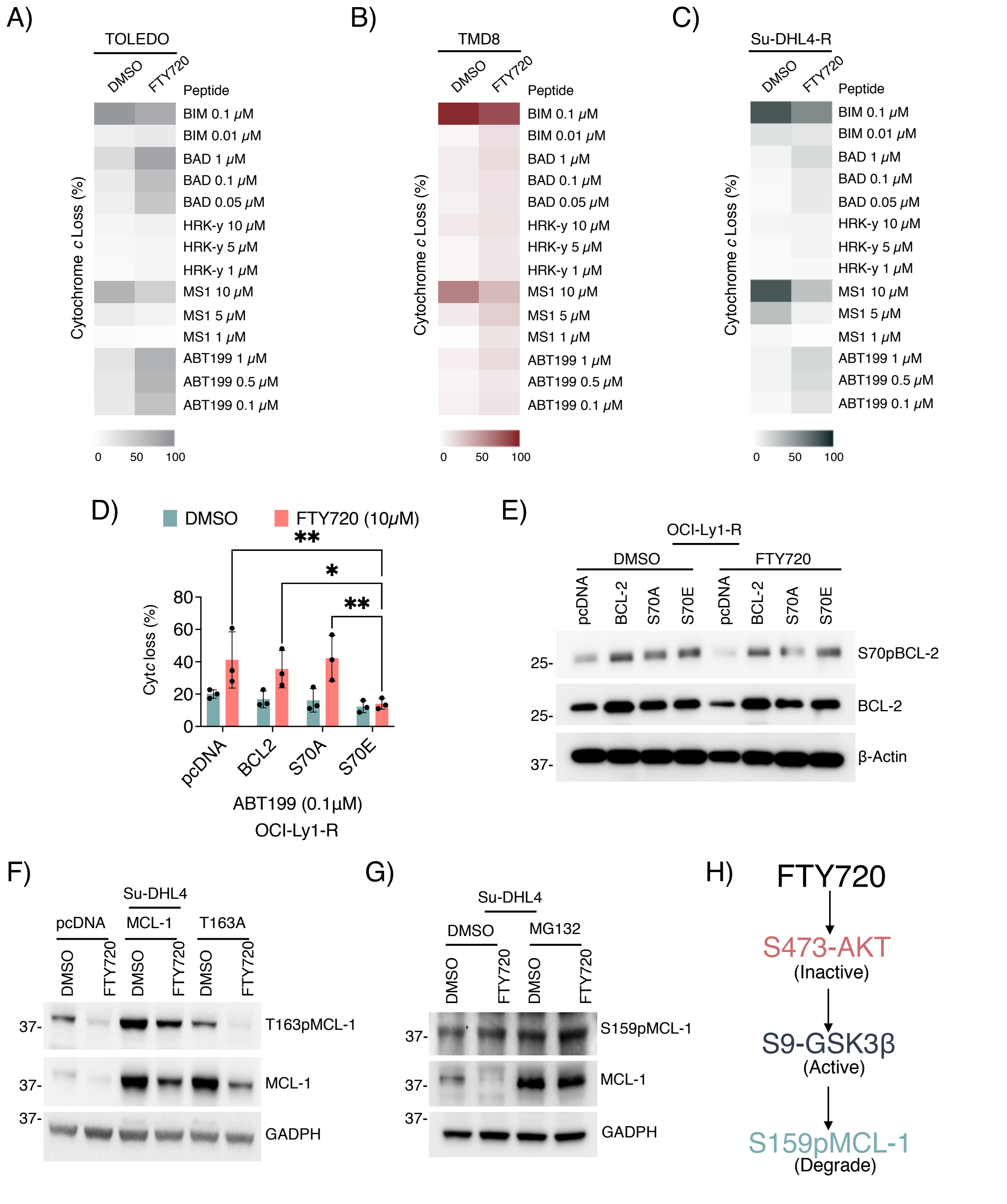

Supplemental Figure 3. FTY720 re-wires dependence to BCL-2 by reducing BCL-2 family protein phosphorylation.

A-C) DBP of TOLEDO (n=3), TMD8 (n=3) and Su-DHL4-R (n=3) following treatment with FTY720 (10μM) for 4 hours.

D) Measurement of %Cyt*c* loss with ABT199/VEN (0.1μM) following 4-hour FTY720 treatment in pcDNA3.1, wild-type BCL-2, p.S70A or p.S70E transfected OCI-Ly1-R cells. (n=3). Sidak's multiple comparisons test was used.

E) Western blot analysis showing S70pBCL-2, BCL-2 and β-Actin levels following 4-hour FTY720 treatment in pcDNA3.1, wild-type BCL-2, p.S70A and p.S70E transfected OCI-Ly1-R cells.

F) Western blot analysis showing T163pMCL-1, MCL-1 and β-Actin levels following 4-hour FTY720 treatment in pcDNA3.1, wild-type MCL-1 and p.T163A transfected Su-DHL4 cells.

G) Western blot analysis showing the increase in S159pMCL-1 in Su-DHL4 cells following pre-treatment with MG132 (5μM) for 4 hours and co-treatment with FTY720 (10μM) for 4 hours.

H) Diagram showing that FTY720 could inactivate AKT by reducing S473pAKT, leading to downstream activation of GSK3β by reducing S9pGSK3β and subsequent priming of MCL-1 degradation via GSK3β-dependent phosphorylation of MCL-1 at Serine-159 (S159pMCL-1).

Supplementary Figure 4

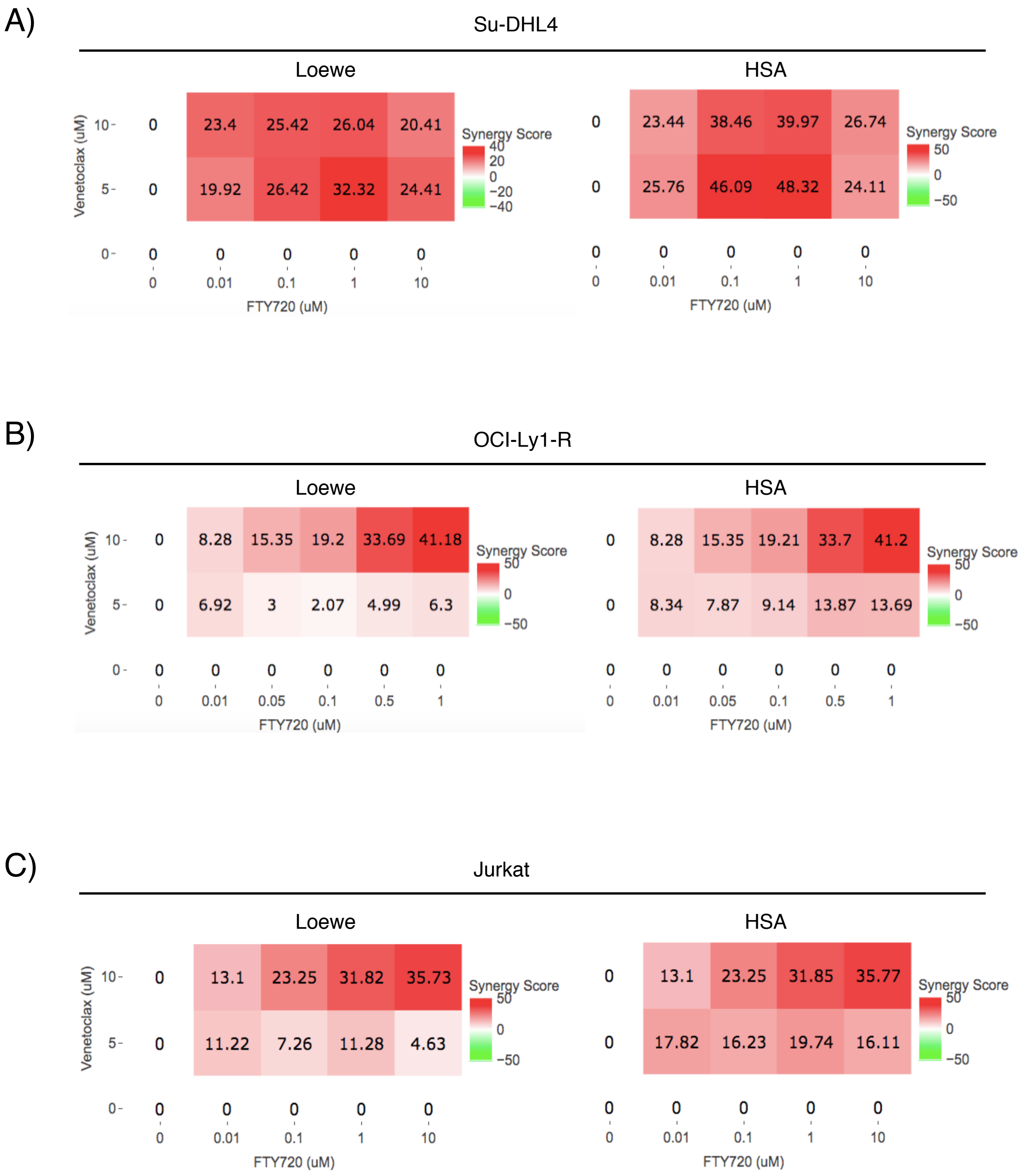

Supplemental Figure 4. Treatment combination of FTY720 and venetoclax display synergism against resistant cells.

A-C) Synergy scores of Su-DHL4, OCI-Ly1-R and Jurkat were computed by SynergyFinder based on Loewe and HSA Synergy models. Red indicates synergism and green indicates antagonism.

Supplemental Figure 5

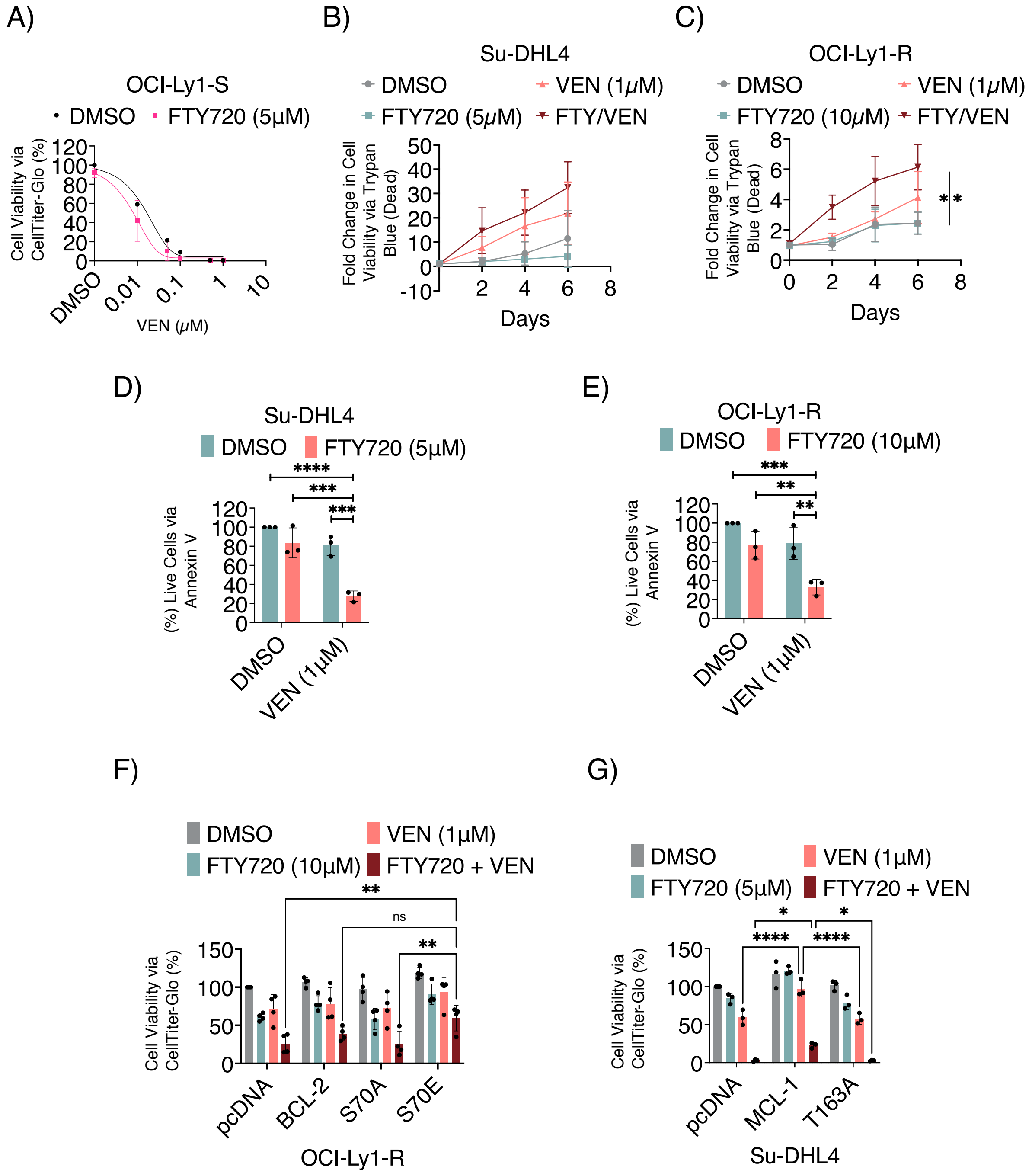

Supplemental Figure 5. FTY720 and venetoclax treatment combination induces apoptotic-cell death.

A) Cell viability of OCI-Ly1-S (n=3) cells following 4-hour pre-treatment with FTY720 and 48-hour increasing concentrations of ABT-199/VEN co-treatment, measured by CTG assay.

B-C) Cell viability of dead Su-DHL4 (n=3) or OCI-Ly1-R (n=4) cell counts following 4-hour pre-treatment with FTY720 and subsequent co-treatment with venetoclax at a time chase of 2,

4 and 6 days, measured by Trypan Blue Exclusion assay. Drug concentrations used were based on the highest/best synergy score. Tukey's multiple comparisons tests were used.

D-E) Cell viability of Su-DHL4 (n=3) or OCI-Ly1-R (n=3) cells following 4-hour pre-treatment with FTY720 and subsequent 48-hour co-treatment with venetoclax, measured by Annexin V/Hoechst assay. Sidak's multiple comparisons tests were used.

F) Cell viability of pcDNA3.1, wild-type BCL-2, p.S70A or p.S70E transient transfected OCI-Ly1-R cells following pre-treatment with FTY720 (10μM) for 4 hours and subsequent co-treatment with venetoclax (1μM) for 48 hours, measured by CTG assay. (n=4). Dunnett's multiple comparisons test was used.

G) Cell viability of pcDNA3.1, wild-type MCL-1 or p.T163A transient transfected Su-DHL4 cells following pre-treatment with FTY720 (5μM) for 4 hours and subsequent co-treatment with venetoclax (1μM) for 24 hours, measured by CTG assay. (n=3). Dunnett's multiple comparisons test was used.

Supplementary Figure 6

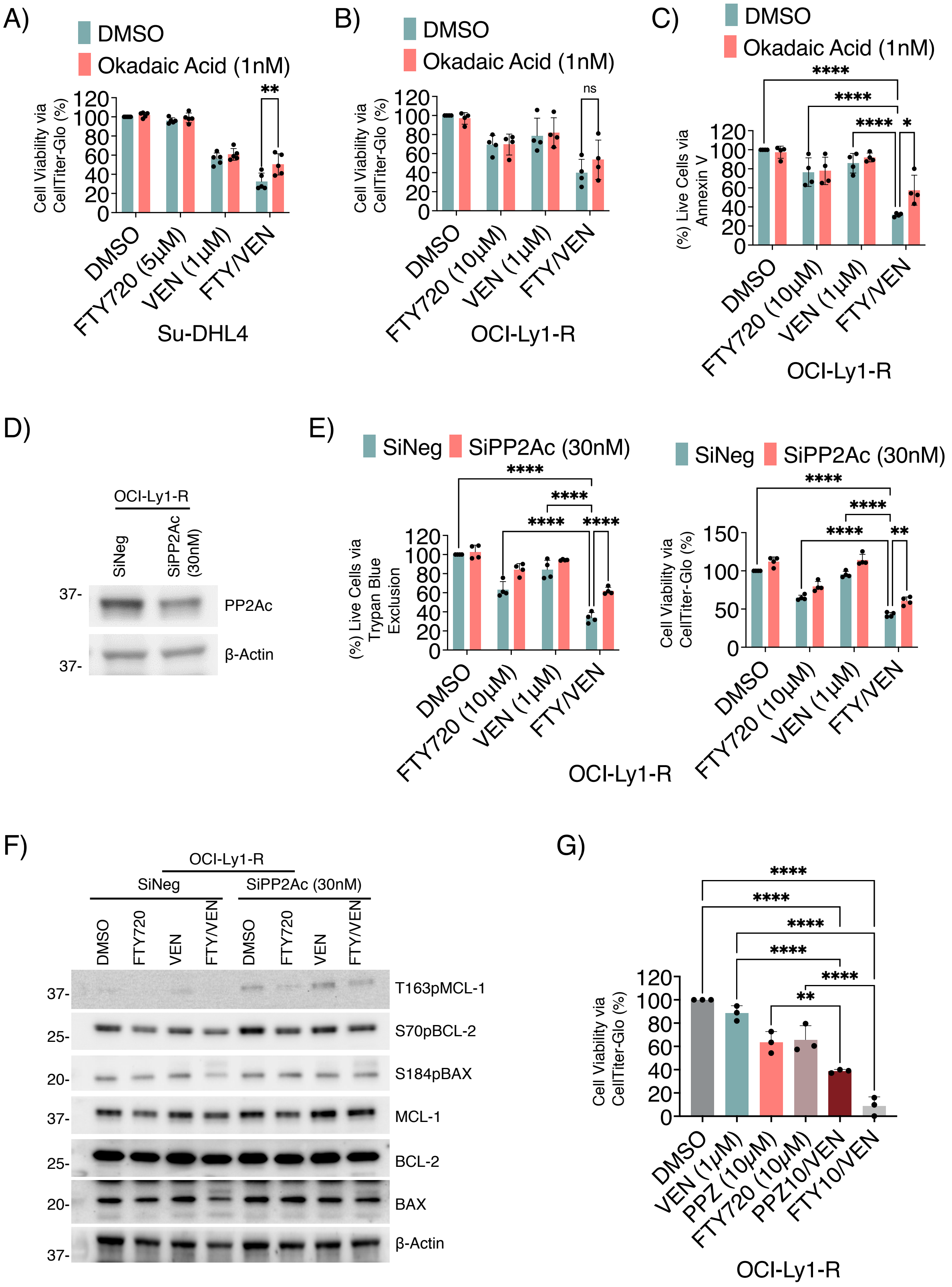

Supplemental Figure 6. Active PP2A is required for the cytotoxic effects of PAD.

A-B) Cell viability of Su-DHL4 (n=5) or OCI-Ly1-R (n=4) cells following pre-treatment with okadaic acid (OA) (1nM) for 2 hours followed by FTY720 (10μM) for 4 hours and subsequent venetoclax (1μM) for 48 hours co-treatment, measured by Cell-Titer Glo (CTG) assay. Sidak's multiple comparisons tests were used.

C) Cell viability of OCI-Ly1-R cells following pre-treatment with OA (1nM) for 2 hours followed by FTY720 (10μM) for 4 hours and subsequent venetoclax (1μM) for 48 hours co-treatment, measured by Annexin/Hoechst assay. (n=4). Sidak's multiple comparisons tests was used.

D) Western blot analysis showing PP2Ac knockdown following siPP2Ac (30nM) treatment for 48 hours.

E) Cell viability of OCI-Ly1-R cells following pre-treatment with siPP2Ac (30nM) for 48 hours followed by FTY720 (10μM) for 4 hours and subsequent venetoclax (1μM) co-treatment for 48 hours, measured by Trypan Blue Exclusion and CTG assays. (n=4). Sidak's multiple comparisons tests were used.

F) Western blot analysis showing reversal of T163pMCL-1, S70pBCL-2, S184pBAX, MCL-1 reductions in OCI-Ly1-R cells following pre-treatment with siPP2Ac (30nM) for 48 hours followed by FTY720 (10μM) for 4 hours and subsequent venetoclax (1μM) co-treatment for 24 hours.

G) Cell viability of OCI-Ly1-R (n=3) cells following pre-treatment with perphenazine (PPZ) or FTY720 for 4 hours followed by venetoclax (1μM) co-treatment for 48 hours, measured by CTG assay. Sidak's multiple comparisons tests were used.

Supplemental Figure 7

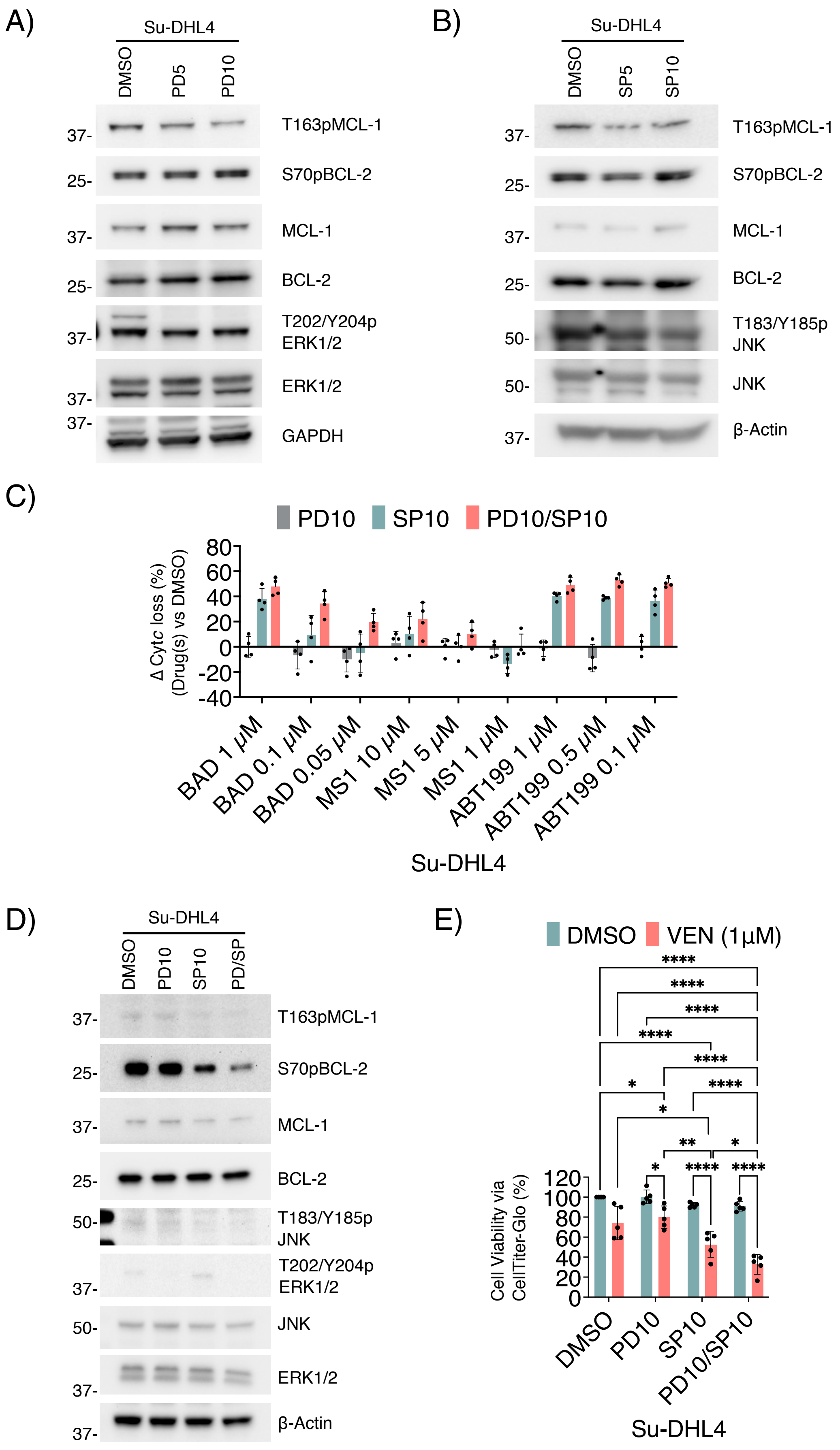

Supplemental Figure 7. Multiple kinases regulate the phosphorylation of BCL-2 family proteins and resistance to venetoclax.

A) Western blot analysis showing unapparent changes of T163pMCL-1, S70pBCL-2, MCL-1 in Su-DHL4 cells following increasing treatment concentrations of the MEK/ERK1/2 inhibitor, PD98059 (5μM and 10μM) for 24 hours. Reduction in T202/Y204pERK1/2 as positive control.

B) Western blot analysis showing unapparent changes of T163pMCL-1, S70pBCL-2, MCL-1 in Su-DHL4 cells following increasing treatment concentrations of the JNK inhibitor, SP600125 (5μM and 10μM) for 24 hours. Reduction in T183/Y185pJNK as positive control.

C) Δ changes of %Cyt*c* loss (mean+SD) between PD98059 (10μM) and/or SP600125 (10μM) and DMSO treatments in the intrinsically resistant Su-DHL4. (n=4).

D) Western blot analysis showing decreased T163pMCL-1 and S70pBCL-2 in Su-DHL4 cells following co-treatment with PD98059 (10μM) and SP600125 (10μM) for 24 hours.

E) Cell viability of Su-DHL4 (n=5) cells following pre-treatment with PD98059 (10μM) and/or SP600125 (10μM) for 4 hours followed by co-treatment with venetoclax (1μM) for 48 hours, measured by CTG assay. Tukey's multiple comparisons tests were used.

Supplemental Figure 8

A)

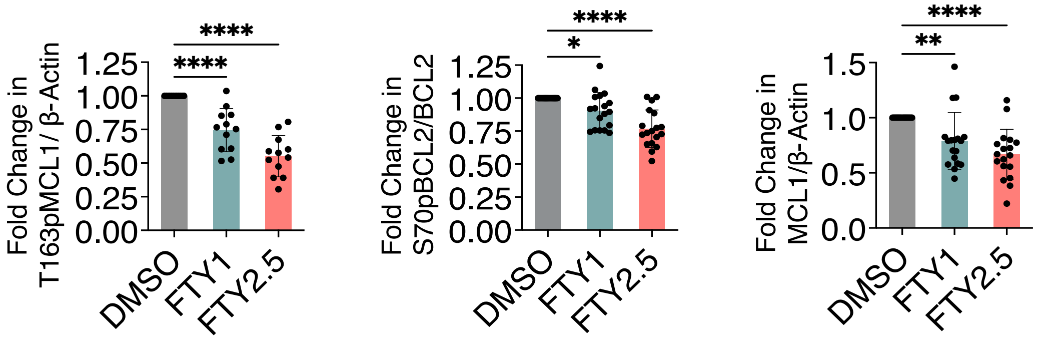

Supplemental Figure 8. FTY720 reduces T163pMCL-1, S70pBCL-2 and MCL-1 in treatment-naïve CLL patient samples.

A) Quantified normalized expression values derived from T163pMCL-1/β-Actin, S70pBCL-2/BCL-2 and MCL-1/β-Actin following ex vivo treatment with FTY720 at 1μM and 2.5μM on CLL cells, 4 hours for figure 12B. Bands quantified by ImageJ software. Dunnett's multiple comparisons tests were used.

Supplementary Figure 9

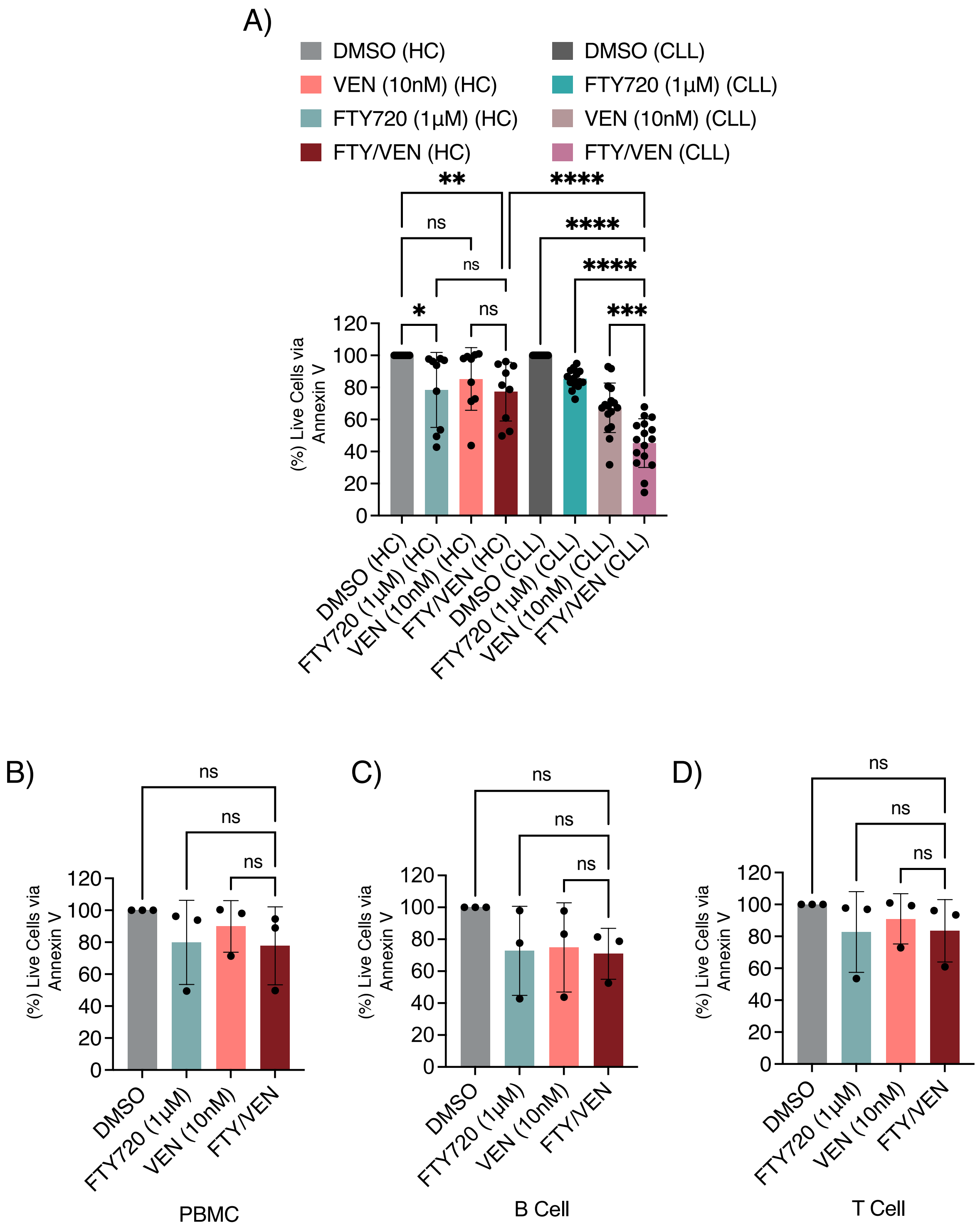

Supplemental Figure 9. FTY720 and venetoclax combination is effective against primary CLL cells but not normal PBMC, T cells and B cells from healthy donors.

A) Cell viability of 9 healthy donor normal primary cells (HC, n=9), consisting of PBMC (n=3), B cells (n=3) and T cells (n=3) and primary CLL cells (CLL, n=16) following pre-treatment with FTY720 (1µM) for 4 hours followed by co-treatment with venetoclax (10nM) for 24 hours, measured by Annexin/Hoechst assay. Sidak's multiple comparisons tests was used. Some DMSO and venetoclax mono-treatment control samples of primary CLL used in this experiment were shared with those used for the cell viability experiment done in figure 12D due to limited patient materials.

B-D) Cell viability of primary cells (same 9 samples from figure S9A), divided to their respective types, PBMC (n=3), B cells (n=3) and T cells (n=3) following pre-treatment with FTY720 (1µM) for 4 hours followed by co-treatment with venetoclax (10nM) for 24 hours, measured by Annexin/Hoechst assay. Sidak's multiple comparisons tests was used.

Supplemental Figure 10

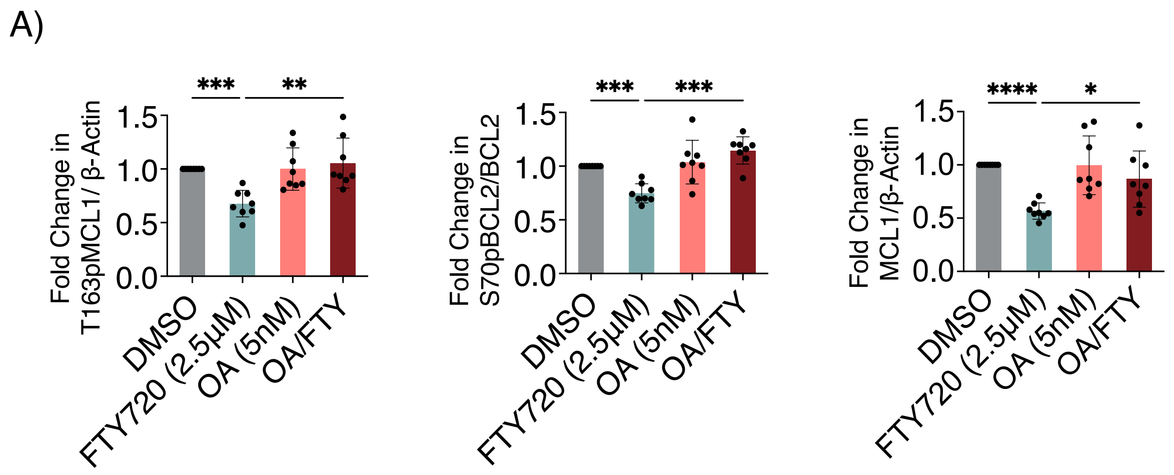

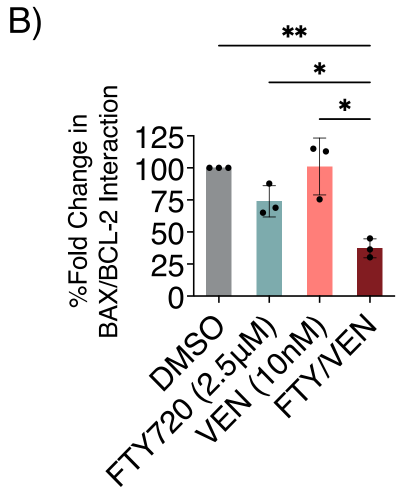

Supplemental Figure 10. Ex vivo treatment with FTY720 reduces BCL-2 family protein phosphorylation via PP2A activation and subsequently affect the pro- and anti-apoptotic protein interaction in treatment-naïve CLL patient samples.

A) Quantified normalized expression values derived from T163pMCL-1/β-Actin, S70pBCL-2/BCL-2 and MCL-1/β-Actin following ex vivo pre-treatment with OA (5nM) for 2 hours followed by co-treatment with FTY720 (2.5µM) for 4 hours in CLL cells for figure 12F. Bands quantified by ImageJ software. Sidak's multiple comparisons tests were used.

B) Densitometry analyses of % normalized BAX/BCL-2 pull-down following ex vivo 4-hour pre-treatment with FTY720 (2.5µM) and 1-hour co-treatment with ABT-199/VEN (10nM) in CLL cells for figure 12G. Dunnett's multiple comparisons test was used.

Supplemental Figure 11

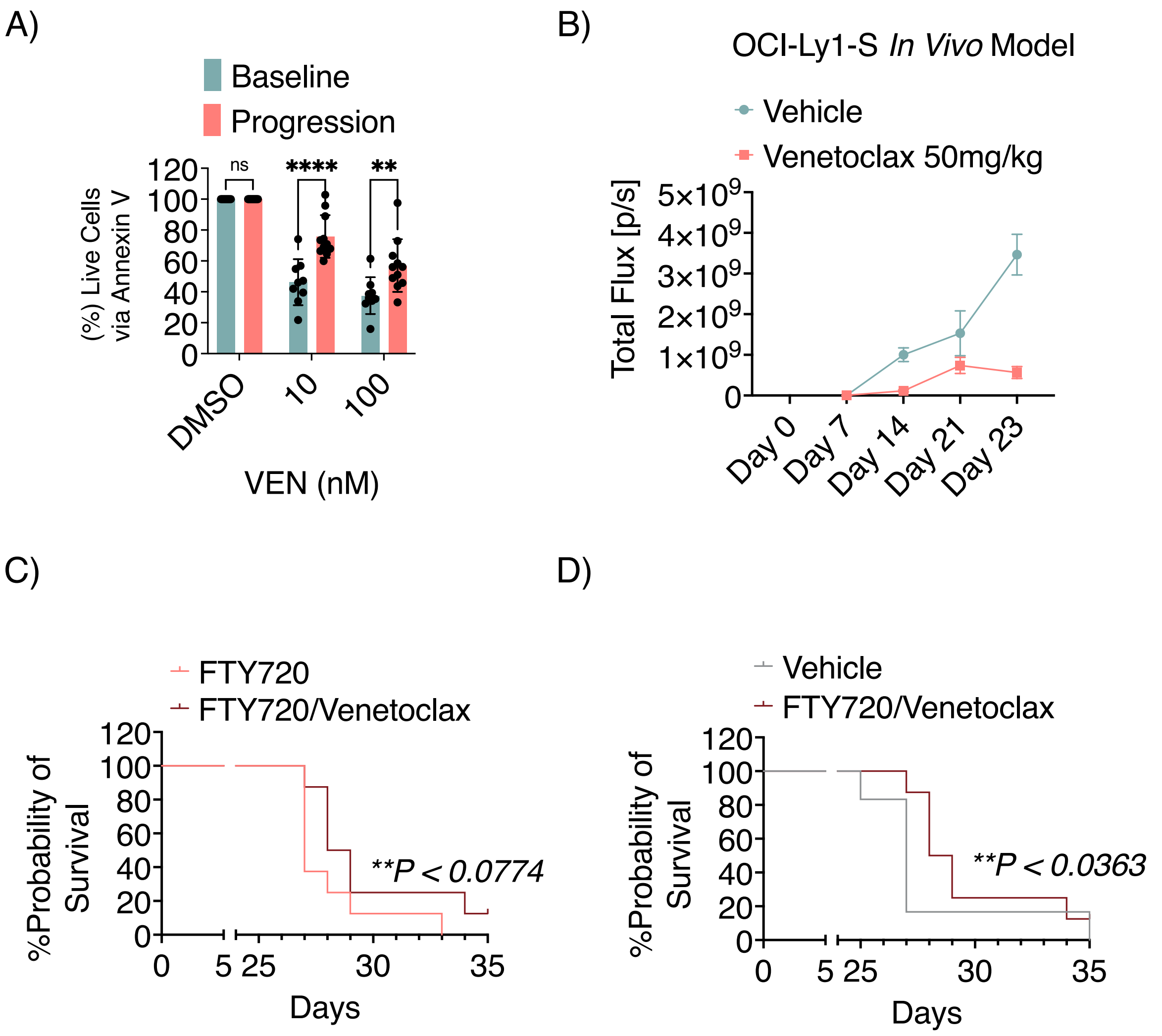

Supplemental Figure 11. FTY720 re-sensitized the resistant phenotype of venetoclax in vivo.

A) Cell viability of paired venetoclax-pretreatment/baseline (*n=9) and venetoclax progression primary CLL (*n=11) cells following ex vivo treatment with venetoclax (VEN) at 10nM and 100nM for 24 hours, measured by Annexin V/Hoechst assay. (Difference in n due to limited patient materials). Sidak's multiple comparisons test was used.

B) Quantified total flux (p/s) of BLI (mean ± SEM) for luciferase-tagged OCI-Ly1-S (n=8 per arm) implanted NSG mice with venetoclax (50mg/kg) daily and vehicle treatments at different timepoints.

C) %Survival probability for luciferase-tagged OCI-Ly1-R implanted NSG mice treated with either FTY720 or FTY720 and venetoclax combination. Gehan-Breslow-Wilcoxon test was used.

D) %Survival probability for luciferase-tagged OCI-Ly1-R implanted NSG mice treated with either vehicle or FTY720 and venetoclax combination. Gehan-Breslow-Wilcoxon test was used.

Supplementary Tables

Supplemental Table 1

Subject ID	IGHV Mutational Status	FISH Results	Complex Karyotype[1]	*TP53* Status
**01**	Mutated	del(13q)	No	Unmutated
**02**	Unmutated	del(13q)	Not avail.	Unmutated
**03**	Mutated	del(13q), del(17p)	No	Unmutated
**04**	Unmutated	Normal karyotype	No	Unmutated
**05**	Mutated	del(13q)	No	Unmutated
**06**	Mutated	Normal karyotype	No	Unmutated
**07**	Mutated	del(13q)	No	Unmutated
**08**	Not avail.	Normal karyotype	Not avail.	Unmutated
**09**	Mutated	del(13q)	No	Unmutated
**10**	Mutated	del(13q)	No	Unmutated
**11**	Mutated	del(13q)	No	Unmutated
**12**	Not avail	del(13q)	No	Unmutated
**13**	Mutated	Normal karyotype	No	Unmutated
**14**	Unmutated	trisomy 12	No	Unmutated

**15**	Unmutated	del(13q)	No	Unmutated
**16**	Mutated	del(13q)	Not avail.	Unmutated
**17**	Mutated	del(13q)	Not avail.	Unmutated
**18**	Mutated	del(13q)	Not avail.	Unmutated
**19**	Unmutated	del(13q)	No	Unmutated
**20**	Unmutated	del(13q), del(11q)	No	Unmutated
**21**	Mutated	del(13q)	No	Unmutated
**22**	Unmutated	del(13q)	No	Unmutated
**23**	Mutated	del(13q)	No	Unmutated
**24**	Unmutated	Normal karyotype	No	Unmutated
**25**	Mutated	del(13q)	No	Unmutated
**26**	Unmutated	trisomy 12	No	Unmutated
**27**	Mutated	trisomy 12	No	Unmutated
**28**	Mutated	del(13q)	No	Unmutated

[1]Complex karyotype defined as ≥ 3 cytogenetic abnormalities.

FISH, fluorescence in situ hybridization. Avail., available. n = 28 patient samples.

Supplemental Table 1. CLL Disease Characteristics of Venetoclax-Untreated/Treatment-Naïve Patient Samples.

Supplemental Table 2

**Subject ID**	**IGHV Mutational Status**	**FISH Results**	**Complex Karyotype[1]**	***TP53* Status**	**Time On Ven Prior to PD (mos)**
**A**	Unmutated	del(13q), del(6q)	No	Not avail.	51.0
**B**	Not avail.	del(13q)	Yes	Mutated	4.0
**C**	Mutated	del(17p)	Yes	Mutated	28.7
**D**	Unmutated	del(13q), del(11q)	No	Unmutated	31.0
**E**	Mutated	del(13q), del(11q)	Yes	Mutated	57.3
**F**	Unmutated	del(13q), del(11q)	Yes	Mutated	22.9
**G**	Mutated	del (13q)	No	Unmutated	23.0
**H**	Unmutated	del (13q)	No	Mutated	30.5
**I**	Unmutated	del(17p)	Yes	Mutated	43.3
**J**	Unmutated	del(13q), del(11q)	Yes	Unmutated	3.2
**K**	Unmutated	del (11q)	Yes	Unmutated	16.8
**L**	Unmutated	del (13q)	Yes	Mutated	7.0
**M**	Unmutated	del(13q), del(6q)	Yes	Not avail.	17.9

[1]Complex karyotype defined as ≥ 3 cytogenetic abnormalities.

FISH, fluorescence in situ hybridization. Ven, venetoclax. PD, progressive disease. Mos, months. Avail., available. n = 13 patient samples.

Supplemental Table 2. CLL Disease Characteristics of Venetoclax-Resistant Patient Samples.

Supplementary Materials and Methods

BH3 profiling technique

The BH3 profiling technique measures the proximity of cells to apoptosis as well as the dependences of cells to specific anti-apoptotic proteins for survival (1, 2). For baseline BH3 profiling, 5 million primary CLL cells derived from the peripheral blood or 3 million cells of immortalized cell line were suspended in 1.65mL MEB2P buffer (150 mM mannitol, 10 mM HEPES-KOH pH 7.5, 150 mM KCl, 1 mM EGTA, 1 mM EDTA, 0.1% BSA, 5 mM succinate, 0.25% poloxamer 188) prior to transferring 15µL of the cell suspension with an automated multi-channel pipette to the BH3 profiling 384-well plate, which consist of 0.002% digitonin for cell membrane permeabilization, increasing concentrations of BIM, BAD, PUMA, HRK and MS1 11-mer pro-apoptotic peptides (New England Peptide, MA, USA) as well as BH3 mimetic ABT199 (venetoclax). Cells were incubated for 1 hour in this plate followed by fixation with 15µL of 4% paraformaldehyde for 30 minutes and then neutralization with 15µL of N2 buffer (1.7 M Tris, 1.25 M Glycine pH 9.1) for 20 minutes. Primary cells were then stained with 10µL of a staining cocktail consisting the PE-Cy7-conjugated CD19 (#302216, Biolegend), PE-conjugated CD5 (#300608, Biolegend), Alexa-488-conjugated cytochrome *c* (#612308, Biolegend) antibodies and Hoechst 33342 (H3570, Invitrogen) and incubated overnight prior to analysis on the BD FACS Fortessa flow cytometry. Cells were gated in the order of Hoechst as live cells, CD5+ and CD19+ for CLL primary cell surface markers for the Alexa-488-conjugated cytochrome *c* signal. A similar cocktail without PE-Cy7-conjugated CD5 and PE-conjugated CD19 was used for cell lines. For Dynamic BH3 profiling (DBP), cells were first treated with drug-of-interest prior to initiating the BH3 profiling as mentioned in this method. Cytochrome *c* release was computed as the degree of MOMP induction to identify anti-apoptotic protein dependences in response to each pro-apoptotic peptide following the normalization to 0% cytochrome *c* release by DMSO (negative control) and 100% cytochrome *c* release by alamethicin (positive control, #SC-200094A). Induction/increase in

cytochrome *c* release, which indicates specific dependences for anti-apoptotic protein, by HRK is measured for BCL-xL, MS1 for MCL-1, BAD for BCL-2 and/or BCL-xL and venetoclax for BCL-2. BIM was used as overall mitochondrial priming for apoptosis.

Kinase activity mapping assay

To measure the activity of kinases in biological samples, we used a biochemical assay named high throughput kinase-activity mapping (HT-KAM) platform (3). HT-KAM uses libraries of peptides as sensors of the phosphorylation activity of kinases (3). The activity of kinase enzymes is derived from their respective subset of biological peptide targets included in the assay. Peptides are derived from computationally curated biological targets of kinases' substrates deposited in PhosphoAtlas (4). The phospho-catalytic signature of cell extracts is established from simultaneously occurring ATP-consumption tests measured in the presence of individual peptides that are experimentally isolated from each other. Assays are run in 384 or 1536 well-plates handled by automated liquid-dispensing instruments. Each experimental well contains one peptide (200µg/ml 11-mer peptide), and all wells receive kinase assay buffer (#9802, Cell Signaling Technology), ATP (#9804, Cell Signaling Technology), cell sample (prepared from cell at ~10µg/ml total protein extract). All reagents are kept on ice and plates on cold blocks until enzymatic reactions are started. Once the dispensing of the reaction mixtures is complete, plates are incubated for 1h at 30ºC. ATP is detected using Kinase-Glo revealing reagent (#V3772, Promega), which stops the activity of the kinases and produces a luminescent signal that directly correlates with the amount of remaining ATP in the samples. For a more detailed description of the peptide sensors design, sequence and connectivity between peptides and kinases, as well as data normalization steps and analysis, refer to: (3, 5).

Here, we used HT-KAM as a modular peptide-sensor platform to specifically interrogate the activity of kinases that can affect the phosphorylation and stability of BCL-2 family proteins, i.e., we chose to measure and compare the activity of kinases belonging to the MAPK, PKA, PKC, PKD, CDK and AMPK families (the complete list of kinases and kinase families is provided in Fig. 4E).

To prepare protein extracts to run on the HT-KAM platform, cells were washed three times with cold PBS and lysed with freshly prepared 1X cell lysis buffer (#9803, Cell Signaling Technology) complemented with 1X of Halt Protease & Phosphatase (#1861281, Thermo

Fisher Scientific). Cell lysates were collected and spun down at 14,000rpm for 15min at 4ºC and supernatants stored at -80ºC.

Cell viability assays

i) CellTiter-Glo® cell viability assay

Drug-treated cells were harvested and seeded in 96-well flat-bottom white plate (#3922, Corning). CellTiter-Glo® 2.0 (CTG) solution (#G9242, Promega) was added to the samples at a 1:1 ratio prior to measurement using the SpectraMax M3 microplate reader (Molecular Devices).

ii) Trypan Blue exclusion assay

Live and dead drug-treated cells were measured using the Trypan blue stain (#T10282, Invitrogen) and Countess® II cell counter (#AMQAX1000R, Invitrogen).

iii) Annexin V/Hoechst assay

Stroma NKTert cell co-culture was used to sustain survival for primary cells at a 1:10 (NKTert:CLL) ratio. NKTert cells were first seeded in 24-well plate 24 hours prior to addition of primary cells and drug treatment. Following drug treatment, cells were harvested and seeded in 96-well flat-bottom plates and stained with Annexin V and Hoechst staining in 1x Annexin-V binding buffer (100 mM HEPES, 40 mM KCl, 1.4 M NaCl, 7.5 mM $MgCl_2$, 25 mM $CaCl_2$ pH 7.4) at room temperature (RT) for 15 minutes. Samples were then fixed with Annexin V fix buffer (4% parafolmaldehyde, 0.5% glutaraldehyde in 1X Annexin V binding buffer) at RT for 10 minutes and neutralized with N2 buffer (1.7 M Tris, 1.25 M glycine, pH 9.1) prior to analysis using the BD FACS Fortessa flow cytometry as previously described (6). Stroma NKTert cells were not used for cell lines.

Generation of figure diagrams

Figure diagrams were generated by BioRender or Microsoft Powerpoint software as listed in figure legends.

Western Blot Analysis and densitometry quantification

Cells were lysed in RIPA buffer (#R0278, Sigma-Aldrich) supplemented with protease and phosphatase inhibitors (#A32961, ThermoFisher Scientific). Cell lysates containing 30-50μg proteins (measured by Pierce™ BCA Protein assay kit, #23225) were mixed with loading dye

(#7722, Cell Signaling), heated at 95°C for 10 minutes and subjected to the XCell SureLock™ Mini-Cell electrophoresis (#EI0002, Invitrogen) and iBlot™ (#IB21001S, Invitrogen) systems. Precision Plus Protein™ Kaleidoscope™ prestained protein standards (#1610375, Bio-Rad) was used. Protein membranes were then developed using the Chemidoc Imaging System (#17001401, Bio-Rad) and protein band densitometry quantification was performed using ImageJ software. All western blot analyses were repeated twice for reproducibility and representative images of the blots were shown in figures.

Plasmids, SiRNAs and Transfection

BCL-2 p.S70A and p.S70E mutant plasmids were previously generated from *pcDNA3.1-hBCL2* plasmid. *MCL-1* p.T163A mutant plasmid was generated from *pcDNA3.1-hMCL-1* plasmid. ON-TARGETplus PPP2CA siRNA (L-003598-01-0020) and non-targeting pool (D-001810-10-20) were purchased from Horizon Discovery. 10 million cells were transfected with 5µg plasmid or 75nM siRNA using the Neon™ Electroporator (#MPK5000, Thermo Fisher Scientific) as previously described (6, 7).

Co-immunoprecipitation (Co-IP)

Cells were lysed in Pierce™ IP buffer (#87788, Thermo Fisher Scientific) supplemented with protease and phosphatase inhibitors (#A32961, Thermo Fisher Scientific). Cell lysate containing 1-1.5mg proteins were transferred for pre-clearing using protein agarose A (#sc-2001, Santa Cruz Biotechnology) or G (#sc-2002, Santa Cruz Biotechnology) beads on a rotator at 4°C for 1.5 hours. Supernatant from this pre-clear was then transferred to a new 1.5 mL Eppendorf tube and 3µg IP antibody was added to sample for overnight incubation on a rotator at 4°C. 25µL protein agarose A or G beads were then added to sample for pull-down on a rotator at 4°C for 6 hours. Sample was then washed with Pierce™ IP buffer 3 times before 25µL of loading dye (#7722, Cell Signaling Technology) was added to the sample and heated at 95°C for 15 minutes for Western blot analysis.

Cell lines and Reagents

	**Catalogue No.**	**Supplier**
**Cell lines**		
OCI-Ly1	ACC722	DSMZ
Su-DHL4	CRL-2957	ATCC

TOLEDO	CRL-2631	Wu Lab (DFCI)
TMD8	CVCL_A442	Wu Lab (DFCI)
Jurkat	TIB-152	ATCC
Stroma-NKTert	RCB2350	Riken BRC
**Antibodies**		
AKT	SC-81434	Santa Cruz Biotechnology
Alexa-488 anti-Cytochrome c	612308	BioLegend
AMPK	2793S	Cell Signaling Technology
Anti-mouse IgG, HRP-linked Antibody	7076S	Cell Signaling Technology
Anti-rabbit IgG, HRP-linked Antibody	7074P2	Cell Signaling Technology
B56α	SC-271151	Santa Cruz Biotechnology
B56**δ**	SC-81605	Santa Cruz Biotechnology
BAD	9292	Cell Signaling Technology
BAX	MA514003	Thermo Fisher Scientific
BAK	3814	Cell Signaling Technology
BCL-2	658702	BioLegend
BCL-2 IP	12789-1-AP	Proteintech
BIM	2993	Cell Signaling Technology
BIM IP	SC-374358	Santa Cruz Biotechnology
ERK1/2	4695S	Cell Signaling Technology
GAPDH	5174S	Cell Signaling Technology
JNK	9252	Cell Signaling Technology
MCL-1	559027	BD Biosciences
MCL-1	94296S	Cell Signaling Technology
PE anti-human CD5	300608	BioLegend
PE/Cy7 anti-human CD19	302216	BioLegend
PP2Ac	05421MI	EMD Milipore
S112pBAD	9291S	Cell Signaling Technology
S159pMCL-1	ab111574	Abcam
S184pBAX	PA539778	Thermo Fisher Scientific
S473pAKT	9271S	Cell Signaling Technology

S70pBCL-2	2827S	Cell Signaling Technology
S9pGSK3β	9336S	Cell Signaling Technology
T163pMCL-1	14765S	Cell Signaling Technology
T172pAMPK	2531S	Cell Signaling Technology
T183, Y185pJNK	4668	Cell Signaling Technology
T202, Y204pERK1/2	4370S	Cell Signaling Technology
β-Actin	SC-47778	Santa Cruz Biotechnology
**Plasmids**		
pcDNA3.1	SP Lab	National University of Singapore
Wild Type MCL-1	SP Lab	National University of Singapore
T163A MCL-1	SP Lab	National University of Singapore
Wild Type BCL-2	SP Lab	National University of Singapore
S70A BCL-2	SP Lab	National University of Singapore
S70E BCL-2	SP Lab	National University of Singapore
**SiRNA**		
SiNegative	D-001810-10-20	Horizon Discovery
SiPP2Ac	L-003598-01-0020	Horizon Discovery
**Drugs**		
Alamethicin	#SC-200094A	Santa Cruz Biotechnology
FTY720	SML0700	Sigma-Aldrich
Perphenazine	P6402	Sigma-Aldrich
MG132	S2619	Selleckchem
Okadaic Acid	O9381	Sigma-Aldrich
PD98059	9900L	Cell Signaling Technology
S63845	HY-100741	MedChemExpress
SP600126	S1460	Selleckchem
Venetoclax/ABT198	HY-15531	MedChemExpress

Venetoclax/ABT199	S8048	Selleckchem
**Reagents and Materials**		
Annexin V	A13201	Thermo Fisher Scientific
BH3 peptides	Not Applicable	New England Peptide
Cell-Titer Glo®	G9242	Promega
Hoechst	H3570	Thermo Fisher Scientific
Invitrogen™ Countess™ cell counting chamber slides	C10312	Invitrogen/ Thermo Fisher Scientific
Loading Dye	7722	Cell Signaling Technology
PEG400	S6705	Selleckchem
PHOSAL 50PG	NC0130871	Thermo Fisher Scientific
Pierce™ IP Buffer	87788	Thermo Fisher Scientific
Precision Plus Protein™ Kaleidoscope™ prestained protein standards	1610375	Bio-Rad
Protease and Phosphatase Inhibitors	A32961	Thermo Fisher Scientific
Protein A Beads	sc-2001	Santa Cruz Biotechnology
Protein G Beads	sc-2002	Santa Cruz Biotechnology
RIPA Buffer	R0278	Sigma-Aldrich
Trypan blue stain	T10282	Invitrogen/ Thermo Fisher Scientific

Additional Supplementary References

1. J. Ryan, J. Montero, J. Rocco, A. Letai, iBH3: simple, fixable BH3 profiling to determine apoptotic priming in primary tissue by flow cytometry. *Biol Chem* **397**, 671-678 (2016).
2. V. Del Gaizo Moore, A. Letai, BH3 profiling--measuring integrated function of the mitochondrial apoptotic pathway to predict cell fate decisions. *Cancer Lett* **332**, 202-205 (2013).
3. J. P. Coppé, M. Mori, B. Pan, C. Yau, D. M. Wolf, A. Ruiz-Saenz, D. Brunen, A. Prahallad, P. Cornelissen-Steijger, K. Kemper, C. Posch, C. Wang, C. A. Dreyer, O. Krijgsman, P. R. E. Lee, Z. Chen, D. S. Peeper, M. M. Moasser, R. Bernards, L. J. van

't Veer, Mapping phospho-catalytic dependencies of therapy-resistant tumours reveals actionable vulnerabilities. *Nat Cell Biol* **21**, 778-790 (2019).

4. A. Olow, Z. Chen, R. H. Niedner, D. M. Wolf, C. Yau, A. Pankov, E. P. Lee, L. Brown-Swigart, L. J. van 't Veer, J. P. Coppé, An Atlas of the Human Kinome Reveals the Mutational Landscape Underlying Dysregulated Phosphorylation Cascades in Cancer. *Cancer Res* **76**, 1733-1745 (2016).
5. M. Kim, J. Park, M. Bouhaddou, K. Kim, A. Rojc, M. Modak, M. Soucheray, M. J. McGregor, P. O'Leary, D. Wolf, E. Stevenson, T. K. Foo, D. Mitchell, K. A. Herrington, D. P. Muñoz, B. Tutuncuoglu, K. H. Chen, F. Zheng, J. F. Kreisberg, M. E. Diolaiti, J. D. Gordan, J. P. Coppé, D. L. Swaney, B. Xia, L. van 't Veer, A. Ashworth, T. Ideker, N. J. Krogan, A protein interaction landscape of breast cancer. *Science* **374**, eabf3066 (2021).
6. S. J. F. Chong, K. Iskandar, J. X. H. Lai, J. Qu, D. Raman, R. Valentin, C. Herbaux, M. Collins, I. C. C. Low, T. Loh, M. Davids, S. Pervaiz, Serine-70 phosphorylated Bcl-2 prevents oxidative stress-induced DNA damage by modulating the mitochondrial redox metabolism. *Nucleic Acids Res*, (2020).
7. S. J. F. Chong, J. X. H. Lai, J. Qu, J. Hirpara, J. Kang, K. Swaminathan, T. Loh, A. Kumar, S. Vali, T. Abbasi, S. Pervaiz, A feedforward relationship between active Rac1 and phosphorylated Bcl-2 is critical for sustaining Bcl-2 phosphorylation and promoting cancer progression. *Cancer Lett* **457**, 151-167 (2019).
